# *Tribulus terrestris* Fruit Extract: Bioactive Compounds, ADMET Analysis, and Molecular Docking with Penicillin-Binding Protein 2a Transpeptidase of Methicillin-Resistant *Staphylococcus epidermidis*

**DOI:** 10.3390/cimb47010052

**Published:** 2025-01-15

**Authors:** Khalid J. Alzahrani

**Affiliations:** Department of Clinical Laboratories Sciences, College of Applied Medical Sciences, Taif University, P.O. Box 11099, Taif 21944, Saudi Arabia; ak.jamaan@tu.edu.sa

**Keywords:** *Tribulus terrestris*, MRSE, extract, GC-MS analysis, druggable characteristics, ADMET, PBP2a inhibitors

## Abstract

*Tribulus terrestris* is a rich source of bioactive molecules and thrives in Mediterranean and desert climate regions worldwide. In this study, *Tribulus terrestris* methanolic HPLC fractions were evaluated for bioactive compounds and PBP2a transpeptidase inhibitors against methicillin-resistant *Staphylococcus epidermidis* (MRSE). Among the collected HPLC fractions, F02 of the methanol extract demonstrated potential activity against MRSE01 (15 ± 0.13 mm), MRSE02 (13 ± 0.21 mm), and MRSE03 (16 ± 0.14 mm) isolates. GC-MS analysis of the F02 fraction identified seventeen compounds. Among seventeen compounds, eight have favorable pharmacokinetics and medicinal chemistry; however, on the basis of in silico high water solubility, high GI absorption, blood–brain barrier non-permeability, lack of toxicity, and potential drug-likeness, 1-ethylsulfanylmethyl-2,8,9-trioxa-5-aza-1-sila-bicyclo[3.3.3]undecane and phthalimide, N-(1-hydroxy-2-propyl), were processed for molecular docking. 1-ethylsulfanylmethyl-2,8,9-trioxa-5-aza-1-sila-bicyclo[3.3.3]undecane formed three hydrogen bonds with Ser-452, Thr-584, and Asn-454 residues of the PBP2a transpeptidase. Similarly, phthalimide, N-(1-hydroxy-2-propyl)-formed four hydrogen bonds with Ser-396, Asn-454, Lys-399, and Ser-452 residues of PBP2a transpeptidase. These two compounds are proposed as novel putative PBP2a transpeptidase inhibitors. Further characterization of compounds extracted from *Tribulus terrestris* may aid in identifying novel PBP2a inhibitory agents for managing MRSE infections.

## 1. Introduction

Plants are a significant source of pharmacologically active compounds that benefit human and animal health. Approximately 50% of the population worldwide relies on traditional medicine [1]. The therapeutic potential of medicinal plants is largely ascribed to their secondary metabolites, including flavonoids, alkaloids, phenolics, and other bioactive compounds [2,3]. These metabolites, often produced in response to microbial infections, have demonstrated in vitro medicinal activity against a wide range of pathogens [4].

Plants used as traditional medicine have been evident for decades. Among these, *Tribulus terrestris* (TT) has been used traditionally in Asia to treat gastrointestinal and cardiovascular problems and urinary tract infections [5]. TT, a widespread plant from the Zygophyllaceae family, is found in tropical regions, including the Mediterranean, Asia, the United States, and Mexico. In Saudi Arabia, TT grows abundantly, particularly in the Southern Hejaz and Eastern Najd regions [6,7]. In Chinese medicine, TT fruits and roots have been used for thousands of years to treat eye infections, respiratory tract infections, and mastitis [8]. Moreover, TT leaves, flowers, and seeds have been utilized in folk medicine for their analgesic, antidiabetic, anti-inflammatory, anticancer, antispasmodic, antibacterial, and other therapeutic purposes [6,7,8,9]. The medicinal potential of TT is due to diverse bioactive phytochemicals, not limited to flavonoids, saponins, terpenoids, tannins, and proteins [10].

The emergence of multidrug-resistant (MDR) bacterial pathogens has necessitated the exploration of alternative antibacterial agents. The limitations of current antibiotics, including issues of efficacy, safety, and cost, keep medicinal plants as an important source of antibacterial compounds. *Staphylococcus epidermidis* is a common skin-associated opportunistic pathogen that frequently causes nosocomial bacteremia and exhibits resistance to multiple antibiotics [11].

It has been reported that *S. epidermidis* is an important gene reservoir that disseminates through horizontal transfer to other bacteria and facilitates virulence and antibiotic resistance [12]. Methicillin-resistant *Staphylococcus epidermidis* (MRSE) possesses resistance to beta-lactam antibiotics due to penicillin-binding protein 2a (PBP2a), a product of the *mecA* gene. PBP2a, with transpeptidase and transglycosylase activities, is critical for cell wall peptidoglycan synthesis in resistant strains [13,14,15,16]. MRSE infections are further complicated by biofilm formation, which enhances bacterial survival and resistance to therapeutic molecules. Despite advancements in antimicrobial therapies, vancomycin remains the primary treatment against MRSE infections [16,17].

Recently, computational approaches have been significantly employed to design and develop medicinal molecules from natural products. Prediction of water solubility, Lipinski’s rule of five, absorption, distribution, metabolism, excretion, and toxicity (ADMET) are useful tools with which to explore the biological and synthetic chemistry, drug-likeness, and toxicity of lead molecules [18,19,20]. Similarly, molecular docking analysis is important in the evaluation of the lead compounds’ conformation with the target active sites. These tools provided insights into medicinal molecules and allowed for the exploration of ligand–receptor interactions [21,22,23]. As far as literature mining is concerned, there is a lack of data on the anti-MRSE and anti-PBP2a potential of the HPLC fractions of TT fruit extract.

This study focuses on the methanolic extract of TT fruits to identify bioactive compounds with anti-MRSE activity. Selected compounds were further analyzed for their potential as PBP2a inhibitors, aiming to uncover druggable molecules for combating MRSE infections.

## 2. Materials and Methods

### 2.1. Tribulus terrestris Fruits Collection

*Tribulus terrestris* fruits were collected and authenticated by the botany expert at Abdul Wali Khan University, Mardan, Pakistan. The TT fruit was deposited with a voucher specimen (24-05/TT). The fruits were properly washed, air-dried, and minced into small pieces [24].

### 2.2. Preparation of TT Extracts

TT extracts were prepared as described [25], with some modifications. Briefly, 100 g of powdered TT fruit was soaked in 500 mL methanol and incubated at room temperature for 30 days. The extract was passed through Whatman paper and concentrated via rotary evaporation at 44 °C to remove the solvent.

### 2.3. Identification of Methicillin-Resistant S. epidermidis (MRSE)

Previously collected *Staphylococcus epidermidis* isolates (n = 15) from the Department of Microbiology, AWKUM, Mardan, Pakistan, were processed for MRSE detection. Gram staining and biochemical tests, including catalase, coagulase, DNase, and cefoxitin disc assays, were performed. For the molecular detection of *S. epidermidis*, DNA was extracted using Chelax 100 resins (Biorad, Hercules, CA, USA). Briefly, Chelax 100 resins were added to sample suspension. The mixture was heated at 95 °C for 10 min, and the resins were separated via centrifugation. The supernatant was used for PCR. Species-specific primers targeting the *rdr* gene (forward: AAGAGCGTGGAGAAAAGTATCAAG; reverse: TCGATACCATCAAAAAGTTGG, 130 bp) were used for *S. epidermidis* detection. *mecA* gene primers (forward: AAAATCGATGGTAAAGGTTGGC; reverse: AGTTCTGCAGTACCGGATTTGC, 533 bp) were used for the detection of methicillin resistance previously described [26].

### 2.4. Antimicrobial Susceptibility Testing of MRSE

The Kirby–Bauer disc diffusion method was employed to evaluate the antibiotic susceptibility of *S. epidermidis* isolates [27]. Bacterial isolates were inoculated onto Muller–Hinton agar plates; and antibiotic discs (Oxoid, Hampshire, UK), including penicillin, augmentin, methicillin, vancomycin, ciprofloxacin, linezolid, erythromycin, gentamicin, tetracycline, azithromycin, and clarithromycin, were used. Methicillin resistance was phenotypically confirmed using a cefoxitin disc. The zone diameter of inhibition was measured in mm and interpreted as per CLSI guidelines [28].

### 2.5. Well Diffusion Assay for Anti-MRSE Activity of Methanolic Fruit Extract

The methanolic extracts were checked against methicillin-resistant *S. epidermidis* [29]. Briefly, an overnight MRSE growth was inoculated on Muller–Hinton agar. The TT dried extract was dissolved in dimethyl sulfoxide (DMSO). A 20 µL plant extract was added into each well and incubated overnight at 37 °C. DMSO was incorporated as a solvent control, while augmentin disc was applied as standard antibiotic control. Inhibitory zones were documented in mm.

### 2.6. TT Fractionation Using Gradient HPLC

Methanolic TT extracts were subjected to gradient HPLC fractionation as described earlier [30]. Solidified TT methanolic extracts were dissolved in a distilled water–methanol mixture (ratio: 1:80) and were fractionated using a C-18 column (PerkinElmer, Shelton, CT, USA). The HPLC system was configured with an isocratic LC pump, solvent manager, degassing, and tubing kit. The system default pressure and fractionation pressure were set at 2000 psi and 1820/1950 psi, respectively. The monochromator wavelength was configured at 250 nm. Multiple HPLC fractions were collected and stored for downstream processing.

### 2.7. Well Diffusion Assay for Anti-MRSE Activity of HPLC Fractions

The anti-MRSE activity of HPLC fractions was assessed using the well diffusion method [29]. Briefly, bacterial cultures were refreshed in nutrient broth and incubated at 37 °C for 2 h. Bacterial suspensions, standardized with 0.5 McFarland, were inoculated onto Muller–Hinton agar. Wells were loaded with HPLC fractions, while DMSO was incorporated as a solvent control, and augmentin was incorporated as a standard antibiotic control. All the plates were incubated overnight at 37 °C, and zone of inhibition was measured in mm. The experiment was run in triplicate.

### 2.8. Identification of Bioactive Compounds

GC-MS (Agilent Technologies, Santa Clara, CA, USA) was used to identify bioactive compounds. Samples were injected via a GC automatic liquid sampler into a column (30 m × 250 µm × 0.25 µm). The ESI source, helium as the mobile phase (1 mL/min), and pressure (8.8085 psi) were set accordingly. The oven’s temperature was adjusted from 70 °C to 270 °C. Data acquisition and mass spectral library searches were conducted using MassHunter software (NIST11.L). The three best hits from each library were documented.

### 2.9. Prediction of Lead Hits

The structural parameters of bioactive molecules were retrieved (https://pubchem.ncbi.nlm.nih.gov/ (accessed on 20 October 2024)). Drug-likeness, medicinal chemistry, and ADMET properties were assessed using online tools such as SWISS-ADME (http://www.swissadme.ch/ (accessed on 1 November 2024)) [19] and ProTox 3.0 (https://tox.charite.de/protox3/ (accessed on 5 November 2024)) [23].

### 2.10. Preparation of PBP2a for Docking Assessment

The PBP2a protein sequence of MRSE was obtained from the UniProt database (Accession ID: A0A5E9MEW2_STAEP). The three-dimensional structure of PBP2a protein was obtained from the Protein Data Bank (PDB ID: AF-A0A5E9MEW2-F1-v4). The protein structure was prepared by removing water molecules, adding Gasteiger charges, and minimizing energy. Docking analysis were conducted using MOE software (2016.08) to evaluate the interactions of ligands with the active site residues of the PBP2a enzyme.

### 2.11. Statistical Analysis

Experimental results were reported as the standard error of the mean.

## 3. Results

### 3.1. Confirmation of MRSE

**Figure 1:**
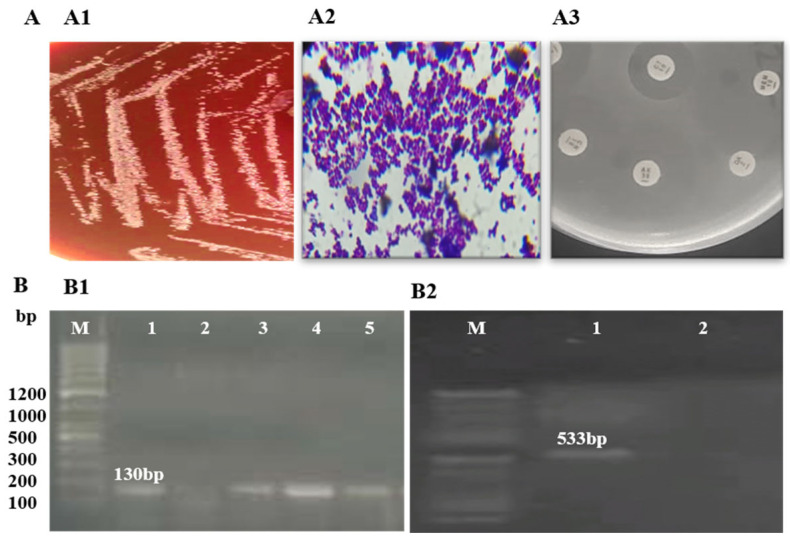
Phenotypic and molecular identification of MRSE: (**A**) phenotypic identification of MRSE; (**A1**) growth on blood agar; (**A2**) Gram staining showing Gram-positive cocci in clusters; (**A3**) antibiotic susceptibility on MHA agar; (**B**) molecular identification of MRSE; (**B1**) amplification of *rdr* (130 bp) for the detection of *S. epidermidis* (M: ladder; 1, 3, 4, 5: *rdr*-positive; 2: *rdr*-negative); (**B2**) amplification of *mecA* (533 bp) (M: ladder; 1: *mecA*-positive; 2: *mecA*-negative).

Identification of *S. epidermidis* isolates was performed using Gram staining and biochemical tests. Amplification of the *rdr* gene, resulting in a 130 bp product on agarose gel electrophoresis, confirmed the identification of *S. epidermidis.* Detection of the *mecA* gene, with a 533 bp amplification product, confirmed methicillin resistance in all three isolates. Further analysis of these MRSE isolates revealed resistance to methicillin, penicillin, augmentin, and erythromycin (Figure 1, Table 1).

### 3.2. HPLC Fractions of TT Methanol Extract Against MRSE

The methanol extract of TT, fractionated via HPLC, was tested for anti-MRSE activity. Among the 15 fractions collected, three fractions demonstrated a zone of inhibition against all MRSE isolates. The F02 fraction exhibited the highest inhibitory activity, with inhibition zones of 15 ± 0.13 mm, 13 ± 0.21 mm, and 16 ± 0.14 mm against MRSE01, MRSE02, and MRSE03, respectively (Table 1). It was noted that the TT fractions exhibited potential activity against MRSE as compared to augmentin (standard antibiotic).

### 3.3. Identification of Bioactive Compounds by GC-MS Analysis

**Figure 2:**
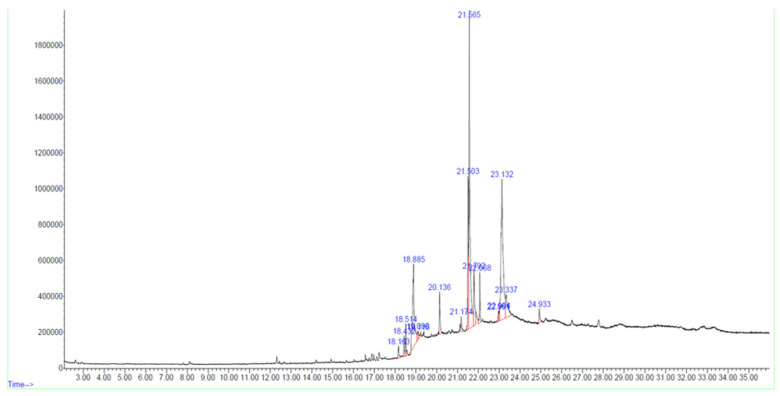
GC-MS chromatogram of bioactive HPLC fraction of *Tribulus terrestris* fruit methanolic extract.

In the F02 fraction, 17 compounds were identified based on spectral library searches. The compounds, along with their molecular formulae, retention times, CAS numbers, m/z ratios, and molecular structures, are detailed in Figure 2, Table 2 and Supplementary Figure S1.

### 3.4. Druggable Characteristics of Bioactive Compounds

The identified 17 compounds were analyzed for druggability using ADMET profiling. Eight compounds, including 13-Hexyloxacyclotridec-10-en-2-one, Bicyclo[5.3.1]undecan-11-one, 13-Oxabicyclo[10.1.0]tridecane, 1-ethylsulfanylmethyl-2,8,9-trioxa-5-aza-1-sila-bicyclo[3.3.3]undecane, N-[[2-p-Tolylsulfonyl]ethyl]phthalimide, Phthalimide, N-(1-hydroxy-2-propyl)-, Oxacyclododecan-2-one, Pyridine-3-carboxamide and oxime, N-(2-trifluoromethylphenyl), exhibited favorable pharmacokinetics, drug-likeness, and medicinal potential (Table 3).

### 3.5. Putative PBP2a Inhibitors

**Figure 3:**
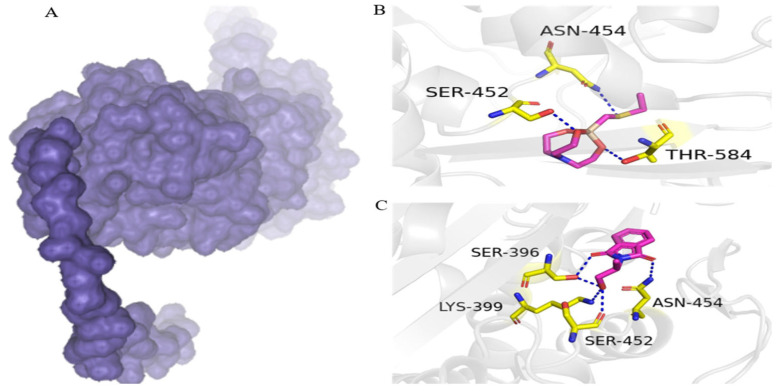
Binding conformation and chemical interaction network of 1-ethylsulfanylmethyl-2,8,9-trioxa-5-aza-1-sila-bicyclo[3.3.3]undecane and phthalimide, N-(1-hydroxy-2-propyl)- within the binding pocket of PBP2a enzyme: (**A**) PBP2a surface; (**B**) interaction map of 1-ethylsulfanylmethyl-2,8,9-trioxa-5-aza-1-sila-bicyclo[3.3.3]undecane with active site residues of PBP2a; (**C**) phthalimide, N-(1-hydroxy-2-propyl)- with active site residues of PB.

On the basis of high water solubility, high GI absorption, blood–brain barrier non-permeability, lack of toxicity, and drug-likeness, two compounds, including 1-ethylsulfanylmethyl-2,8,9-trioxa-5-aza-1-sila-bicyclo[3.3.3]undecane and Phthalimide, N-(1-hydroxy-2-propyl)-, were assessed for molecular interaction with PBP2a transpeptidase of MRSE. 1-Ethylsulfanylmethyl-2,8,9-trioxa-5-aza-1-sila-bicyclo[3.3.3]undecane interacts with Ser-452, Thr-584, and Asn-454 residues of PBP2a transpeptidase via hydrogen bonds. Similarly, phthalimide, N-(1-hydroxy-2-propyl)-formed four hydrogen bonds with PBP2a transpeptidase amino acid residues of Ser-396, Asn-454, Lys-399, and Ser-452 (Figure 3).

## 4. Discussion

The emergence of drug resistance in Gram-positive bacteria, particularly methicillin resistance, has posed significant challenges to the therapeutic efficacy of beta-lactam antibiotics. *Staphylococcus epidermidis* is a well-documented nosocomial pathogen capable of thriving in hospital and community environments. Methicillin-resistant *Staphylococcus epidermidis* (MRSE) expresses PBP2a, which confers resistance to beta-lactam [14]. Given the increasing resistance to conventional antibiotics, the exploration of alternative therapeutic sources, such as medicinal plants, is imperative.

Medicinal plants, including *Tribulus terrestris* (TT), have long been recognized as reservoirs of bioactive compounds with diverse pharmacological activities. TT contains secondary metabolites, such as flavonoids, alkaloids, and steroidal saponins, which contribute to its broad-spectrum antimicrobial, anticancer, and anti-inflammatory properties [8]. Prior studies have demonstrated TT’s efficacy against other bacterial species. For instance, a recent study from Saudi Arabia reported TT’s cytotoxic role against cancer cell lines, as well as its antibacterial and antibiofilm activities against *Streptococcus* and *Lactobacillus* species [31].

No reports to date have evaluated its anti-MRSE potential, which underscores the novelty of the present study. One study identified bioactive molecules in TT leaf ethanolic extracts [32], while another reviewed the phytochemical and pharmacological potential of TT [33]. In this study, methanolic HPLC fractions of TT exhibiting anti-MRSE activity were processed for GC-MS analysis. Seventeen compounds were identified, and most are reported here for the first time in TT fruit.

ADMET analysis revealed that eight compounds showed drug-likeness characteristics and medicinal potential. Among these, 1-ethylsulfanylmethyl-2,8,9-trioxa-5-aza-1-sila-bicyclo[3.3.3]undecane and phthalimide, N-(1-hydroxy-2-propyl)- showed excellent water solubility, high GI absorption, and non-permeability across the blood–brain barrier, fulfilling the five rules of drug-likeness. The compound 1-ethylsulfanylmethyl-2,8,9-trioxa-5-aza-1-sila-bicyclo[3.3.3]undecane was previously identified in *A. chinense* bulb hexane extracts with activity against *Staphylococcus aureus* and *Pseudomonas aeruginosa* [34]; however, it is reported here for the first time in TT fruit methanolic extract. Phthalimide, N-(1-hydroxy-2-propyl) has previously shown anti-inflammatory activity in murine models of chronic inflammation [35].

Docking studies are essential for evaluating the medicinal potential of lead compounds for medicinal potential [21]. These methods allow for the exploration of ligand–receptor affinities and interactions [22]. In this study, 1-ethylsulfanylmethyl-2,8,9-trioxa-5-aza-1-sila-bicyclo[3.3.3]undecane and phthalimide, N-(1-hydroxy-2-propyl)- exhibited multiple interactions with the key residues of the PBP2a transpeptidase enzyme of MRSE. The PBP2a transpeptidase expression in MRSE exhibits resistance to beta-lactam antibiotics [14]. It was found that 1-ethylsulfanylmethyl-2,8,9-trioxa-5-aza-1-sila-bicyclo[3.3.3]undecane and phthalimide, N-(1-hydroxy-2-propyl)- strongly interacted with the PBP2a enzyme; therefore, further studies are recommended to validate their efficacy and safety against MRSE.

In the current study, anti-MRSE methanolic TT fractions were evaluated for bioactive compounds, and processed for ADMET and docking analysis; however, in vitro and in vivo validation will be required to explore the potential and efficacy of the identified compounds. Further characterization of the 1-ethylsulfanylmethyl-2,8,9-trioxa-5-aza-1-sila-bicyclo[3.3.3]undecane and phthalimide, N-(1-hydroxy-2-propyl) will help to elucidate these compounds as new anti-infective agents against MRSE.

## 5. Conclusions

Seventeen compounds were identified in the TT methanolic extract of the F02 fraction, of which eight compounds exhibited favorable druggable characteristics. Among the eight compounds, 1-ethylsulfanylmethyl-2,8,9-trioxa-5-aza-1-sila-bicyclo[3.3.3]undecane and phthalimide, N-(1-hydroxy-2-propyl) were highly water-soluble and made hydrogen bonds with multiple residues of the PBP2a transpeptidase. The findings of this study may open new avenues for natural product research, and they emphasize the need for further preclinical and clinical investigations to validate their therapeutic potential against MRSE and other resistant pathogens.

## Figures and Tables

**Table 1:** Detection and antibiotic susceptibility of MRSE and anti-MRSE activity of *Tribuls terrestris* HPLC methanolic fractions.

Isolates	PCR Detection of MRSE		Antibiotic Susceptibility Profile of MRSE		HPLC Fractions			Antibiotic Control	Solvent Control
	*rdr*	*mecA*	Resistance	Sensitive	Zone of Inhibition (mm)				
					F01	F02	F03	Augmentin	DMSO
MRSE01	Detected	Detected	M, P, AUG, E, CIP	VA, AZM, LIN, TE, CN, CLR	12 ± 0.32	15 ± 0.13	10 ± 0.20	0	0
MRSE02	Detected	Detected	M, P, AUG, CLR, E, CIP	VA, TE, LIN, CN, AZM	10 ± 0.20	13 ± 0.21	9 ± 0.21	0	0
MRSE03	Detected	Detected	M, P, E, AUG, CN, AZM	VA, CLR, CN, LIN, TE, CIP	11 ± 0.10	16 ± 0.14	8 ± 0.20	0	0

Values obtained after deduction of ZOI solvent control and mean ± standard error of the given value. M: methicillin; P: penicillin AUG: augmentin; VA: vancomycin; CN: gentamicin; E: erythromycin; LIN: linezolid; CLR: clarithromycin; AZM: azithromycin; TE: tetracycline; CIP: ciprofloxacin; augmentin: standard antibiotic; DMSO: dimethyl sulfoxide.

**Table 2:** Identification of bioactive compounds from *Tribulus terrestris* fruit methanolic extracts.

S.No.	Compound	Molecular Formula	Ret Time	CAS No.	m/z	2D/3D Structure
1.	13-Hexyloxacyclotridec-10-en-2-one	C_18_H_32_O_2_	18.163	127062-51-5	32.00	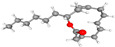
2.	Bicyclo[5.3.1]undecan-11-one	C_11_H_18_O	18.163	013348-11-3	41.10	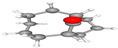
3.	13-Oxabicyclo[10.1.0]tridecane	C_12_H_22_O	18.163	000286-99-7	7.10	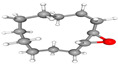
4.	10,13-Octadecadienoic acid, methyl ester	C_19_H_34_O_2_	18.432	056554-62-2	55.10	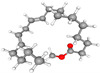
5.	1-Ethylsulfanylmethyl-2,8,9-trioxa-5-aza-1-sila-bicyclo[3.3.3]undecane	C_9_H_19_NO_3_SSi	18.885	063331-02-2	175.10	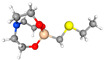
6.	N-[[2-p-Tolylsulfonyl]ethyl]phthalimide	C_17_H_15_NO_4_S	18.885	069384-65-2	314.30	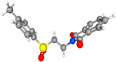
7.	Phthalimide, N-(1-hydroxy-2-propyl)-	C_11_H_11_NO_3_	18.885	1000164-06-1	41.10	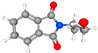
8.	9-Eicosene, (E)-	C_20_H_40_	20.136	074685-29-3	116.00	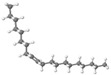
9.	Cyclopropaneoctanal, 2-octyl-	C_19_H_36_O	21.174	056196-06-6	69.10	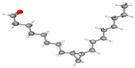
10.	Isopropyl linoleate	C_21_H_38_O_2_	21.503	022882-95-7	55.10	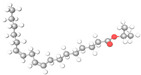
11.	Butyl 9,12-octadecadienoate	C_22_H_40_O_2_	21.503	1000336-54-1	79.10	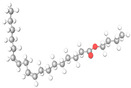
12.	9-Octadecenal, (Z)-	C_18_H_34_O	21.565	002423-10-1	69.10	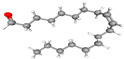
13.	Oxacyclododecan-2-one	C_11_H_20_O_2_	21.792	001725-03-7	129.00	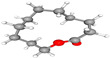
14.	Oleoyl chloride	C_18_H_33_ClO	21.792	000112-77-6	57.10	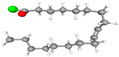
15.	Phthalic acid, di(2-propylpentyl) ester	C_24_H_38_O_4_	22.068	1000377-93-5	41.10	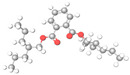
16.	2,3-Dihydroxypropyl elaidate	C_21_H_40_O_4_	23.132	002716-53-2	69.10	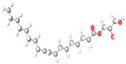
17.	Pyridine-3-carboxamide, oxime, N-(2-trifluoromethylphenyl)-	C_13_H_10_F_3_N_3_O	23.337	288246-53-7	69.10	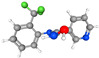

**Table 3:** Druggable compounds of *Tribulus terrestris* fruit methanolic extracts.

Description	Characteristics of Druggable Compounds							
	13-Hexyloxacyclotridec-10-en-2-one	Bicyclo[5.3.1]undecan-11-one	13-Oxabicyclo[10.1.0]tridecane	1-Ethylsulfanylmethyl-2,8,9-trioxa-5-aza-1-sila-bicyclo[3.3.3]undecane	N-[[2-p-Tolylsulfonyl]ethyl]phthalimide	Phthalimide, N-(1-Hydroxy-2-propyl)-	Oxacyclododecan-2-one	Pyridine-3-carboxamide, oxime, N-(2-trifluoromethylphenyl)-
1. Physicochemical Profile								
Chemistry	C_18_H_32_O_2_	C_11_H_18_O	C_12_H_22_O	C_9_H_19_NO_3_SSi	C_17_H_15_NO_4_S	C_11_H_11_NO_3_	C_11_H_20_O_2_	C_13_H_10_F_3_N_3_O
M.W (g/mol)	280.45	166.26	182.30	249.40	329.37	205.21	184.28	281.23 g/mol
Heavy atoms (n)	20	12	13	15	23	15	13	20
Aromatic heavy atoms (n)	0	0	0	0	12	6	0	12
Csp3 (fraction)	0.83	0.91	1.00	1.00	0.18	0.27	0.91	0.08
Rotatable bonds (n)	5	0	0	3	4	2	0	4
Num. H-bond acceptors(n)	2	1	1	4	4	3	2	6
Hydrogen donors (n)	0	0	0	0	0	1	0	2
Refractivity (molar)	87.34	50.96	56.65	66.67	89.11	57.50	54.16	66.47
TPSA (Å^2^)	26.30	17.07	12.53	56.23	79.90	57.61	26.30	57.51
2. Lipophilicity								
iLOGP	4.03	2.26	2.85	2.83	2.28	1.75	2.62	1.99
XLOGP3	6.67	3.12	4.70	0.90	2.20	1.06	3.60	2.36
WLOGP	5.56	2.94	3.67	0.22	2.76	0.28	3.05	4.31
MLOGP	4.17	2.59	3.02	−0.51	2.33	0.90	2.48	2.77
SILICOS-IT	4.69	2.98	3.45	0.15	2.54	1.28	3.01	2.77
Log *P*_o/w_ Consensus	5.03	2.78	3.54	0.72	2.42	1.06	2.95	2.84
3. Water solubility								
ESOL	−5.45	−2.84	−3.93	−1.76	−3.39	−1.94	−3.25	−3.25
Solubility (mg/mL; mol/L)	9.93 × 10^−4^; 3.54 × 10^−6^	2.42 × 10^−1^; 1.46 × 10^−3^	2.14 × 10^−2^; 1.17 × 10^−4^	4.38 × 10; 1.76 × 10^−4^	1.34 × 10^−1^; 4.07 × 10^−4^	2.33 × 10; 1.14 × 10^−4^	1.04 × 10^−1^; 5.62 × 10^−4^	1.58 × 10^−1^; 5.62 × 10^−4^
Solubility class	Moderately soluble	Soluble	Soluble	Very soluble	Soluble	Very soluble	Soluble	Soluble
Log *S* (Ali)	−7.02	−3.15	−4.69	−1.67	−3.51	−1.86	−3.84	−3.21
Solubility (mg/mL; mol/L)	2.65 × 10^−5^; 9.44 × 10^−8^	1.18 × 10^−1^; 7.12 × 10^−4^	3.71 × 10^−3^; 2.03 × 10^−5^	5.38 × 10; 2.16 × 10^−2^	1.01 × 10^−1^; 3.08 × 10^−4^	2.83 × 10; 1.38 × 10^−2^	2.67 × 10^−2^; 1.45 × 10^−4^	1.74 × 10^−1^; 6.20 × 10^−4^
Solubility class	Poorly soluble	Soluble	Moderately soluble	Very soluble	Soluble	Very soluble	Soluble	Soluble
SILICOS-IT	−4.56	−2.35	−2.49	−1.51	−5.67	−2.33	−2.57	−5.00
Solubility (mg/mL; mol/L)	7.72 × 10^−3^; 2.75 × 10^−5^	7.34 × 10^−1^; 4.42 × 10^−3^	5.86 × 10^−1^; 3.21 × 10^−3^	7.62 × 10; 3.05 × 10^−2^	6.96 × 10^−4^; 2.11 × 10^−6^	9.51 × 10^−1^; 4.63 × 10^−3^	4.94 × 10^−1^; 2.68 × 10^−3^	2.84 × 10^−3^; 1.01 × 10^−5^
Solubility class	Moderately soluble	Soluble	Soluble	Soluble	Moderately soluble	Soluble	Soluble	Moderately soluble
4. Pharmacokinetics								
Skin permeability (cm/s)	−3.28	−5.10	−4.08	−7.18	−6.75	−6.80	−4.87	−6.34
Blood brain barrier (permeability)	Yes	Yes	Yes	No	No	No	Yes	Yes
Gastrointestinal absorption	High	High	High	High	High	High	High	High
P-g proteins substrate	No	No	No	No	No	No	No	No
CYP2D6 inhibitor	No	No	No	No	No	No	No	No
CYP2C19 inhibitor	No	No	No	No	Yes	No	No	No
CYP3A4 inhibitor	No	No	No	No	No	No	No	No
CYP2C9 inhibitor	Yes	No	Yes	No	Yes	No	Yes	No
CYP1A2 inhibitor	Yes	No	No	No	No	No	No	No
5. Druglikeness								
Lipinski (violation)	1	0	0	0	0	0	0	0
Ghose	0	0	0	0	0	0	0	0
Veber	0	0	0	0	0	0	0	0
Egan	1	0	0	0	0	0	0	0
Muegge	1	2	2	0	0	0	1	0
Bioavailability (score)	0.55	0.55	0.55	0.55	0.55	0.55	0.55	0.55
6. Medicinal Chemistry								
PAINS (alert)	0	0	0	0	0	0	0	0
Brenk (alert)	1	0	1	1	1	1	0	3
Leadlikeness (violation)	1	1	2	1	0	1	2	0
Synthetic accessibility	4.20	3.17	3.45	5.45	2.48	1.88	2.43	2.63
7. Toxicity								
LD50 predicted (mg/kg)	34,900	500	5000	1800	1250	3500	5000	1500
Toxicity class	6	4	5	4	4	5	5	4
Hepatotoxic	No	No	No	No	No	No	No	No
Nephrotoxic	No	No	No	No	No	No	No	No
Cardiotoxic	No	No	No	No	No	No	No	No
Neurotoxic	No	Yes	No	No	No	No	No	No
Carcinogenic	No	No	Yes	No	No	No	No	No
Cytotoxic	No	No	No	No	No	No	No	No
Mutagenic	No	No	No	No	No	No	No	No
Immunotoxic	No	No	No	No	No	No	No	No

## Data Availability

Data access statements are included in the publication.

## Supplementary Materials

The following supporting information can be downloaded at https://www.mdpi.com/article/10.3390/cimb47010052/s1.
