# Supplementary material for: Tribulus terrestris Fruit Extract: Bioactive Compounds, ADMET Analysis, and Molecular Docking with Penicillin-Binding Protein 2a Transpeptidase of Methicillin-Resistant Staphylococcus epidermidis
Source: Curr Issues Mol Biol. 2025 Jan 15;47(1):52. doi: 10.3390/cimb47010052 (PMC11764108; doi:10.3390/cimb47010052)
Supplement: Supplementary file 1 [file cimb-47-00052-s001.zip › cimb-3375222-supplementary.pdf]

Library Search Report

Data Path : D:\GCMS RESULTS DATA\
Data File : 10619 MUHAMMAD ALI AWKUM.D
Acq On : 16 Oct 2024 23:05
Operator : CRL
Sample : 6/1
Misc :
ALS Vial : 16 Sample Multiplier: 1

Search Libraries: C:\Database\NIST11.L Minimum Quality: 0

Unknown Spectrum: Apex
Integration Events: ChemStation Integrator - events.e

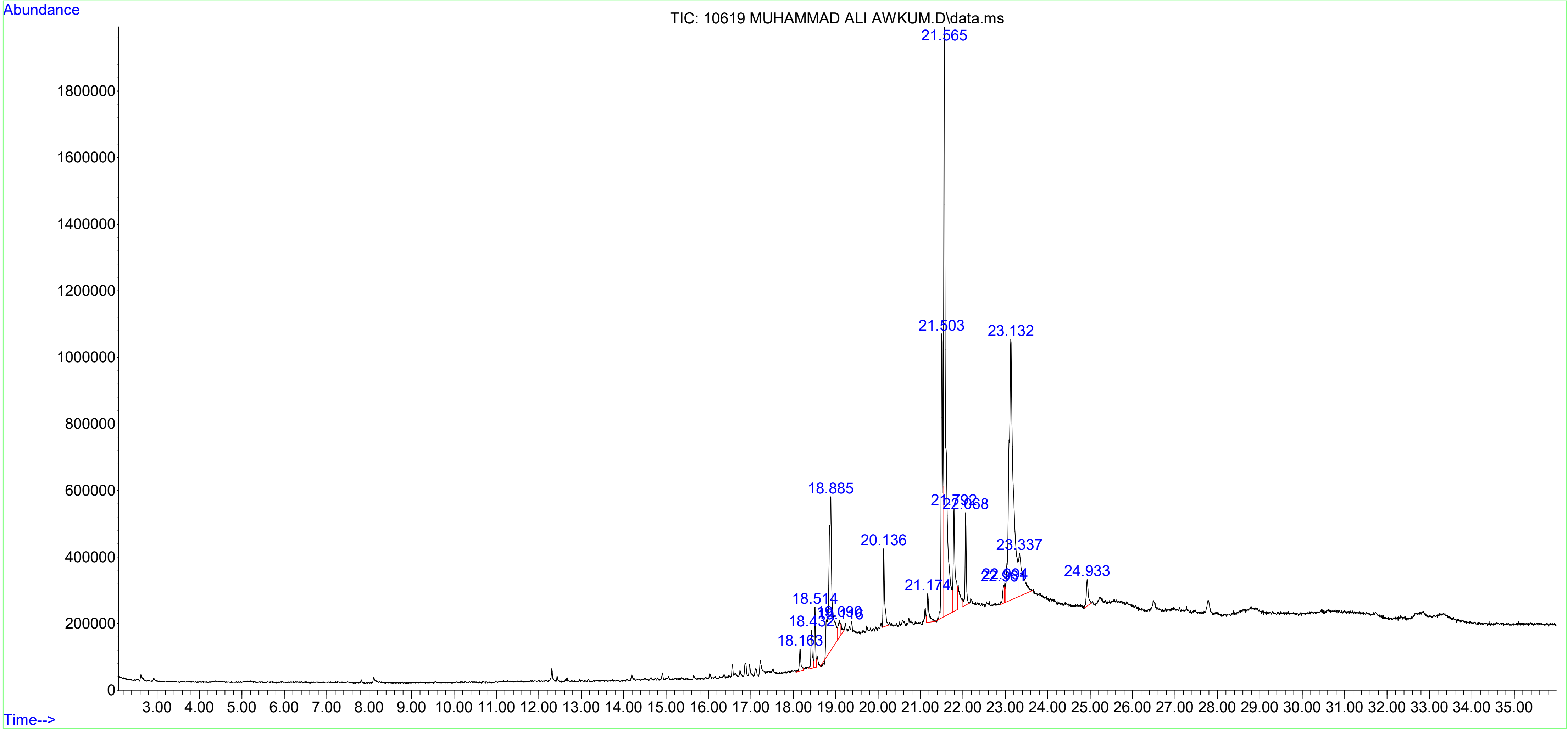

Unknown Spectrum based on Apex

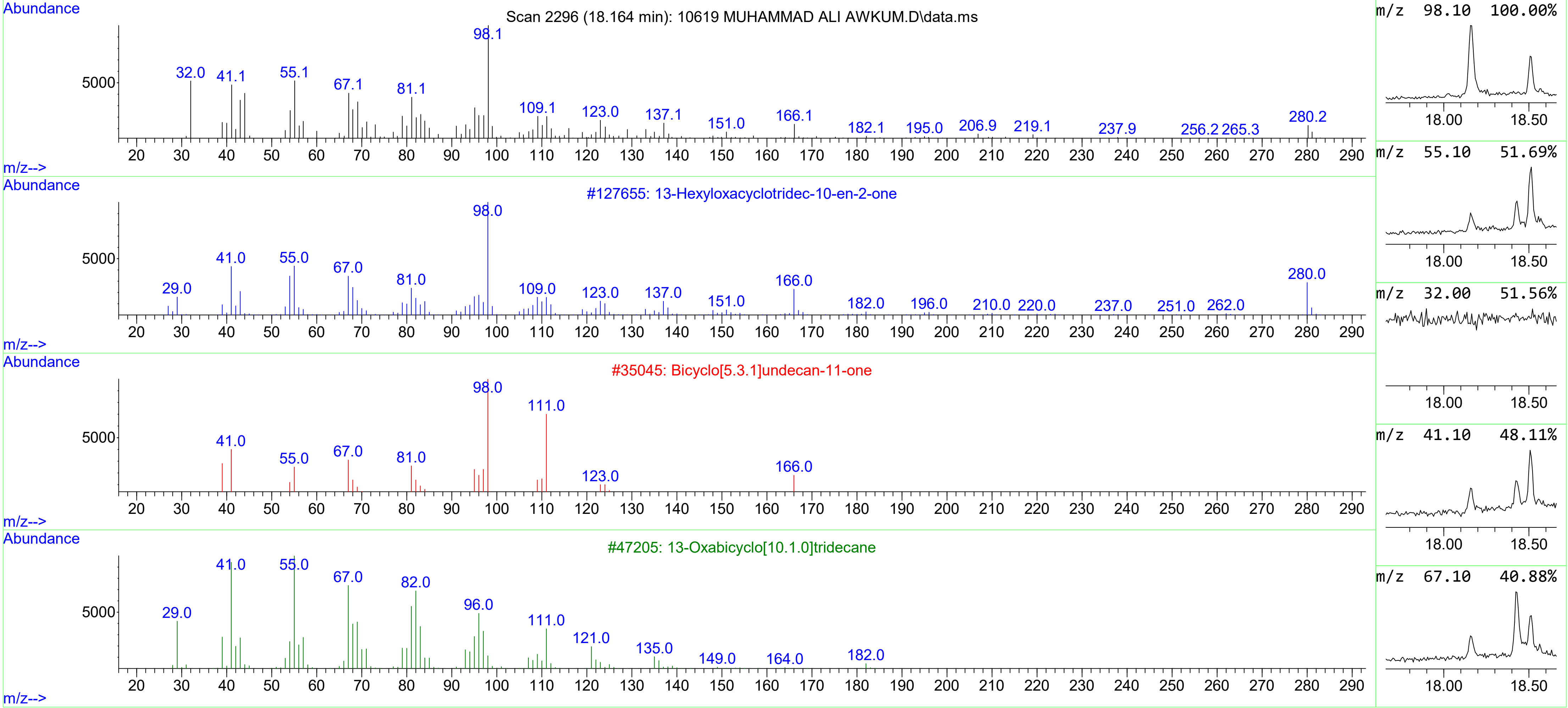

Data File: D:\GCMS RESULTS DATA\10619 MUHAMMAD ALI AWKUM.D
Sample : 6/1

Peak Number: 1 at 18.163 min Area: 1800597 Area % 0.87

The 3 best hits from each library.	Ref\#	CAS\#	Qual
C:\Database\NIST11.L			
1 13-Hexyloxacyclotridec-10-en-2-one	127655	127062-51-5	89
2 Bicyclo[5.3.1]undecan-11-one	35045	013348-11-3	70
3 13-Oxabicyclo[10.1.0]tridecane	47205	000286-99-7	42

Library Search Report - ChemStation Integrator

Unknown Spectrum based on Apex

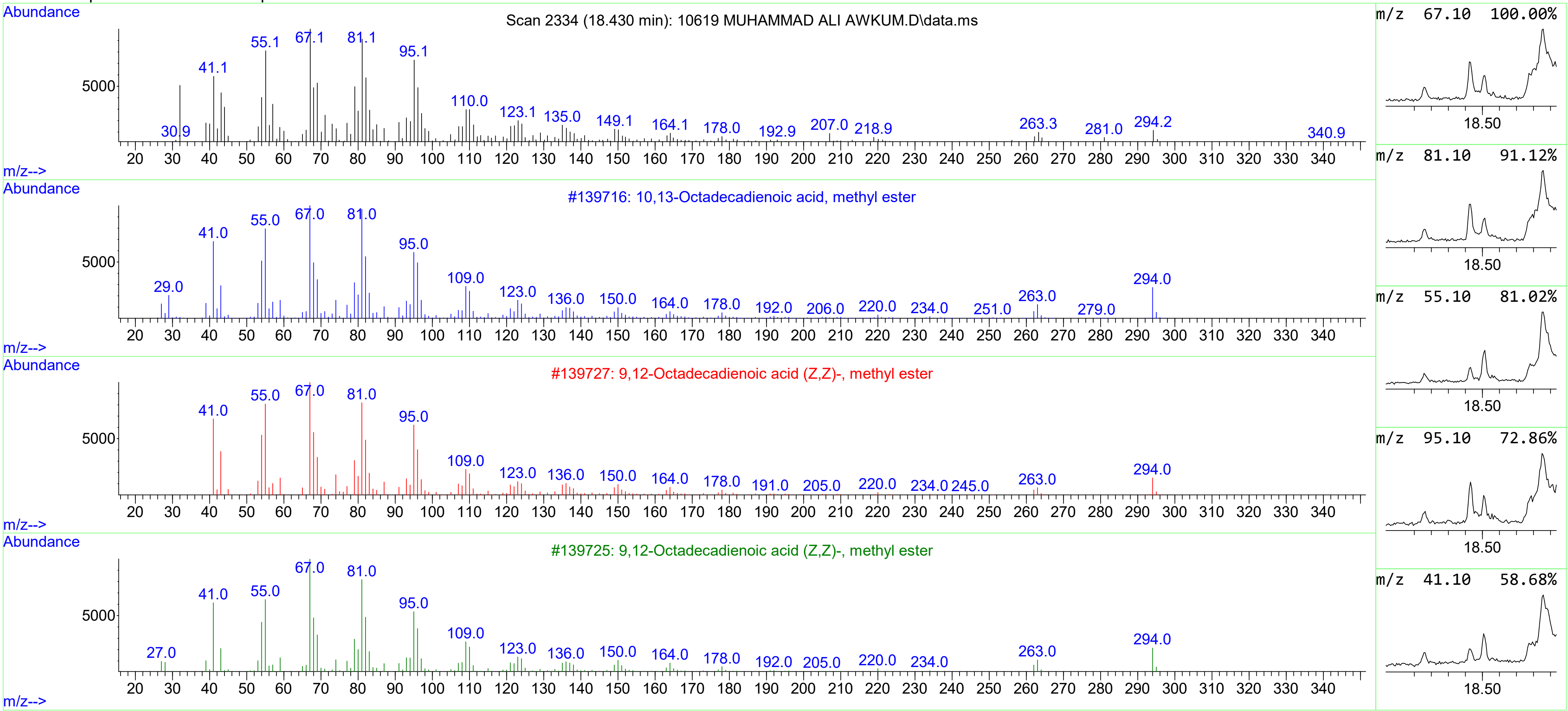

Data File: D:\GCMS RESULTS DATA\10619 MUHAMMAD ALI AWKUM.D
Sample : 6/1

Peak Number: 2 at 18.432 min Area: 2516485 Area % 1.21

The 3 best hits from each library.	Ref#	CAS#	Qual
C:\Database\NIST11.L			
1 10,13-Octadecadienoic acid, meth...	139716	056554-62-2	99
2 9,12-Octadecadienoic acid (Z,Z)-...	139727	000112-63-0	99
3 9,12-Octadecadienoic acid (Z,Z)-...	139725	000112-63-0	99

Unknown Spectrum based on Apex

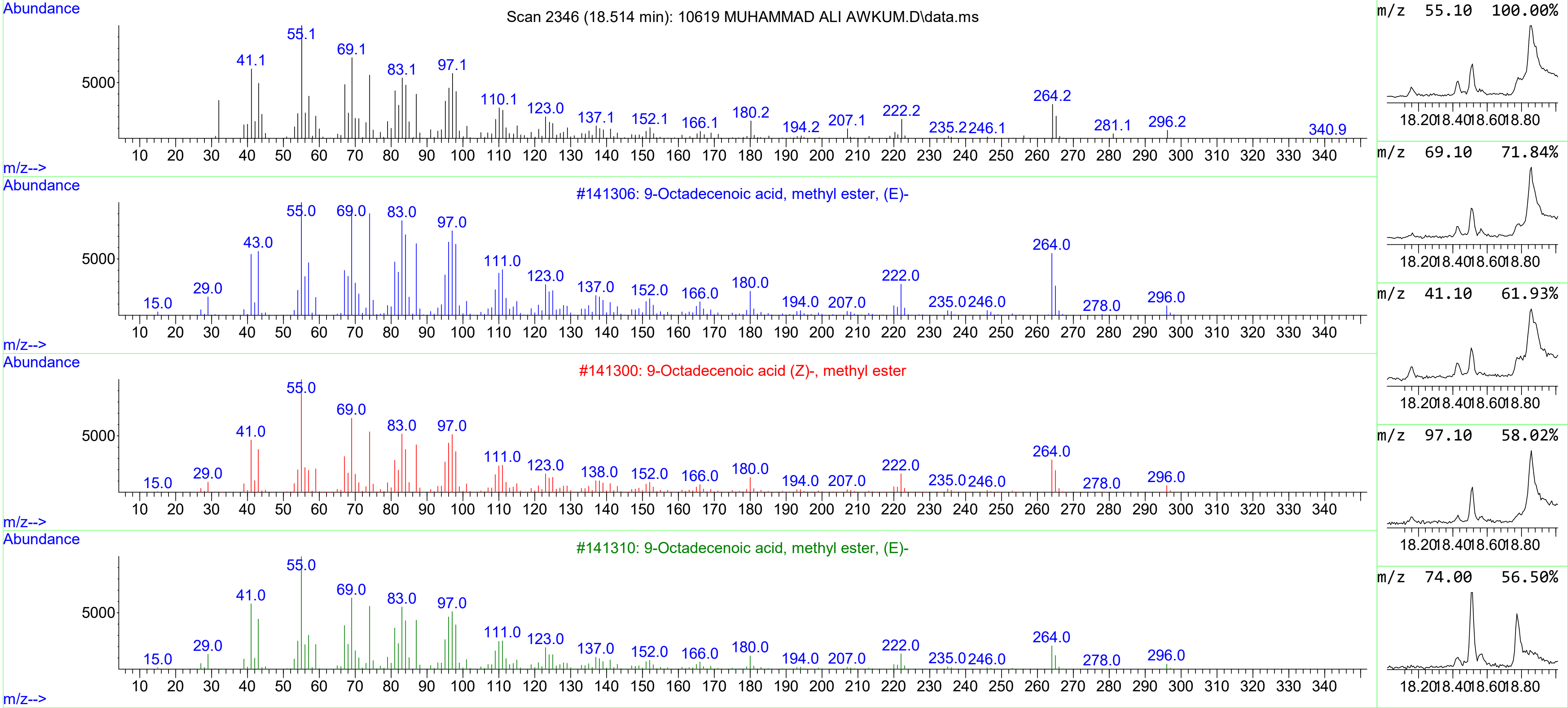

Data File: D:\GCMS RESULTS DATA\10619 MUHAMMAD ALI AWKUM.D
Sample : 6/1

Peak Number: 3 at 18.514 min Area: 3449443 Area % 1.66

The 3 best hits from each library.	Ref\#	CAS\#	Qual
C:\Database\NIST11.L			
1 9-Octadecenoic acid, methyl este...	141306	001937-62-8	99
2 9-Octadecenoic acid (Z)-, methyl...	141300	000112-62-9	99
3 9-Octadecenoic acid, methyl este...	141310	001937-62-8	99

Unknown Spectrum based on Apex

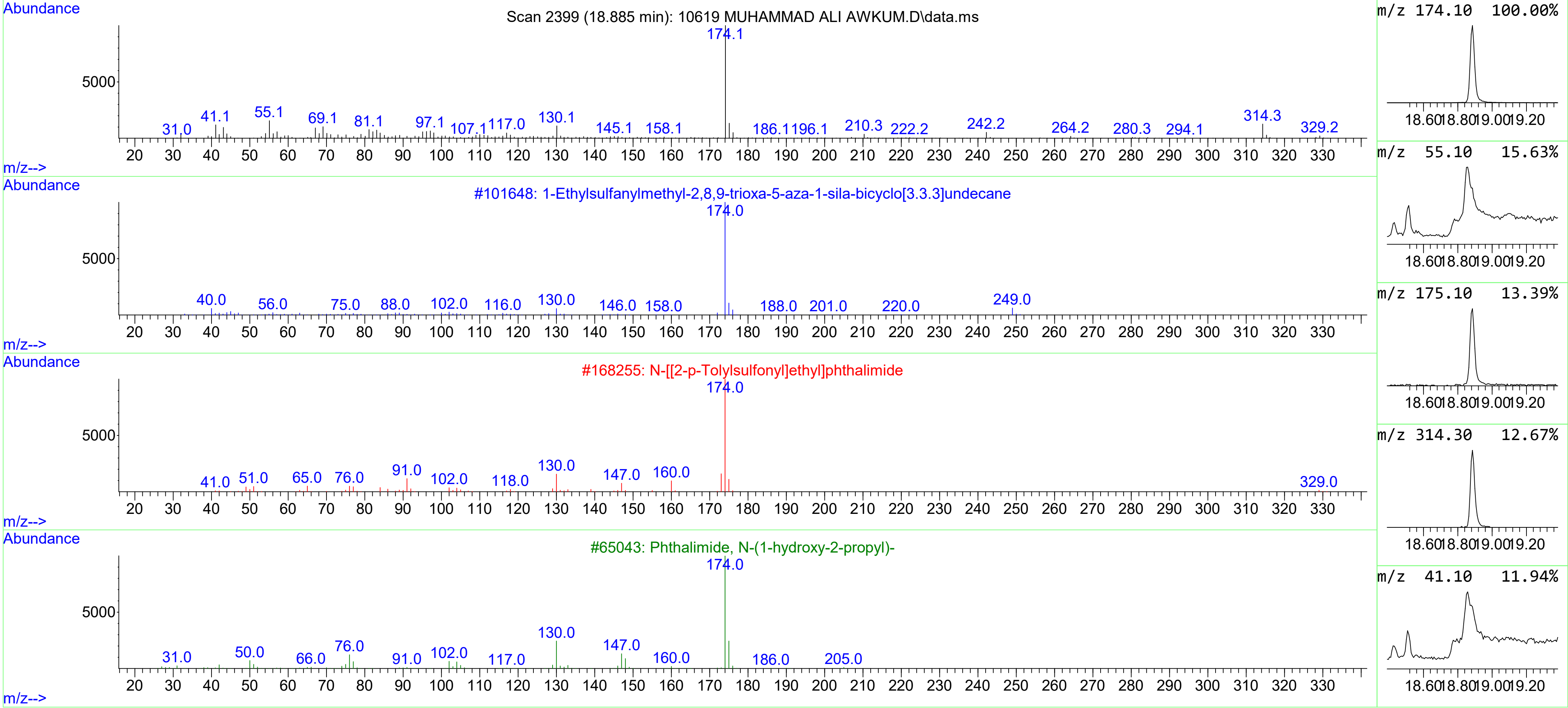

Data File: D:\GCMS RESULTS DATA\10619 MUHAMMAD ALI AWKUM.D
Sample : 6/1

Peak Number: 4 at 18.885 min Area: 25932279 Area % 12.48

The 3 best hits from each library.	Ref\#	CAS\#	Qual
C:\Database\NIST11.L			
1 1-Ethylsulfanylmethyl-2,8,9-trio...	101648	063331-02-2	64
2 N-[[2-p-Tolylsulfonyl]ethyl]phth...	168255	069384-65-2	58
3 Phthalimide, N-(1-hydroxy-2-prop...	65043	1000164-06-1	53

Unknown Spectrum based on Apex

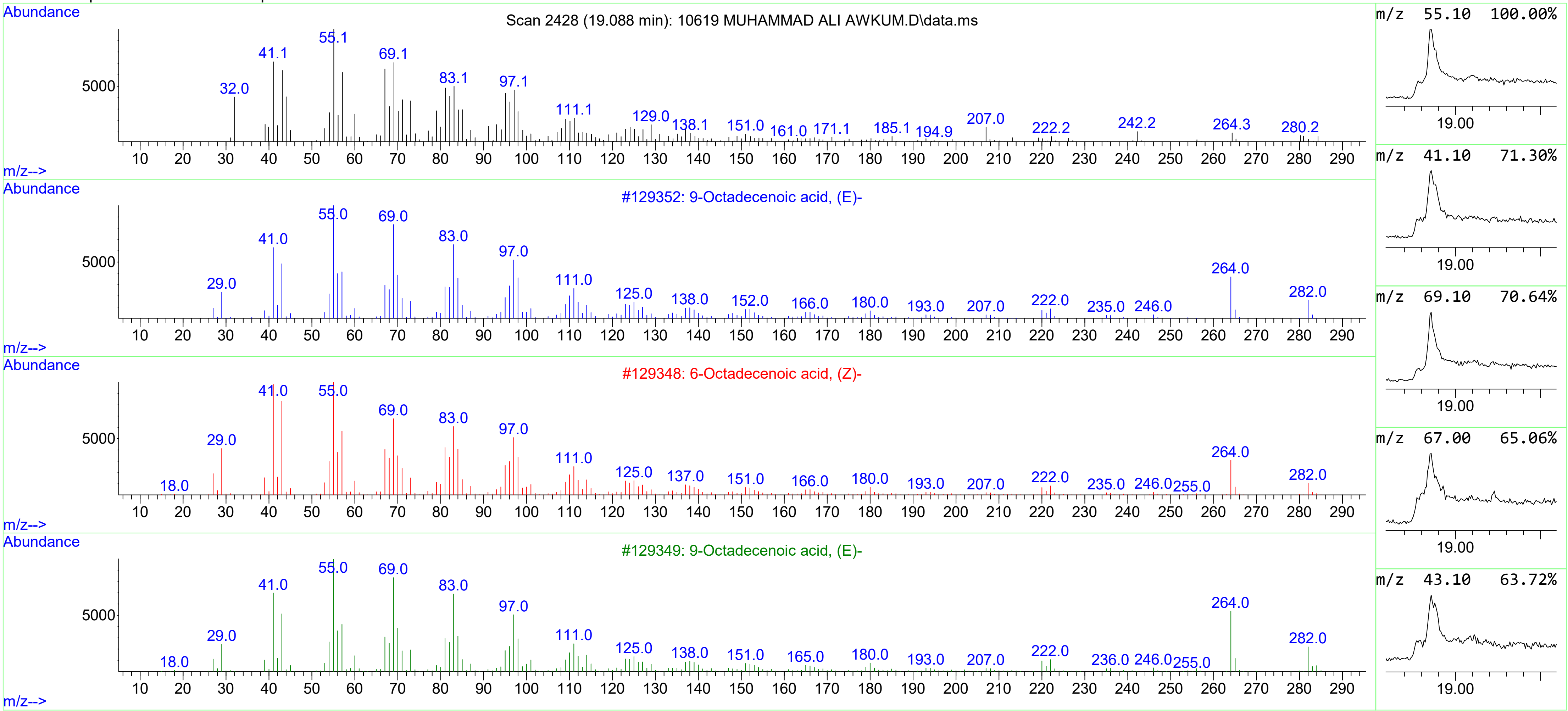


```
Data File: D:\GCMS RESULTS DATA\10619 MUHAMMAD ALI AWKUM.D
Sample   : 6/1

Peak Number:  5     at     19.090 min  Area:    1521978   Area % 0.73

 The 3 best hits from each library.          Ref\#    CAS\#      Qual
---------------------------------------------------------------------
    C:\Database\NIST11.L
     1 9-Octadecenoic acid, (E)-              129352 000112-79-8   99
     2 6-Octadecenoic acid, (Z)-              129348 000593-39-5   98
     3 9-Octadecenoic acid, (E)-              129349 000112-79-8   97
```

Library Search Report - ChemStation Integrator

Unknown Spectrum based on Apex

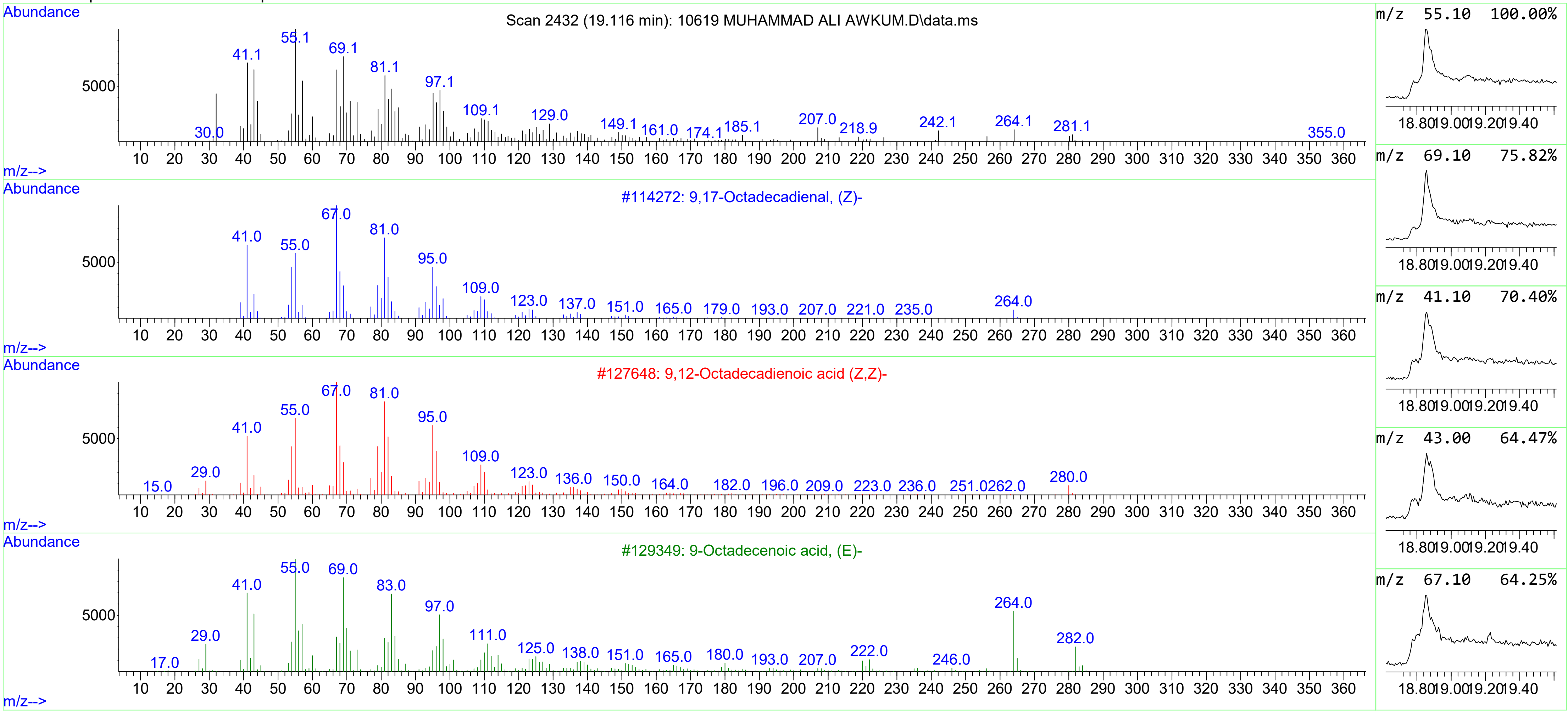

Data File: D:\GCMS RESULTS DATA\10619 MUHAMMAD ALI AWKUM.D
Sample : 6/1

Peak Number: 6 at 19.116 min Area: 992925 Area % 0.48

The 3 best hits from each library.	Ref#	CAS#	Qual
C:\Database\NIST11.L			
1 9,17-Octadecadienal, (Z)-	114272	056554-35-9	95
2 9,12-Octadecadienoic acid (Z,Z)-	127648	000060-33-3	92
3 9-Octadecenoic acid, (E)-	129349	000112-79-8	89

Unknown Spectrum based on Apex

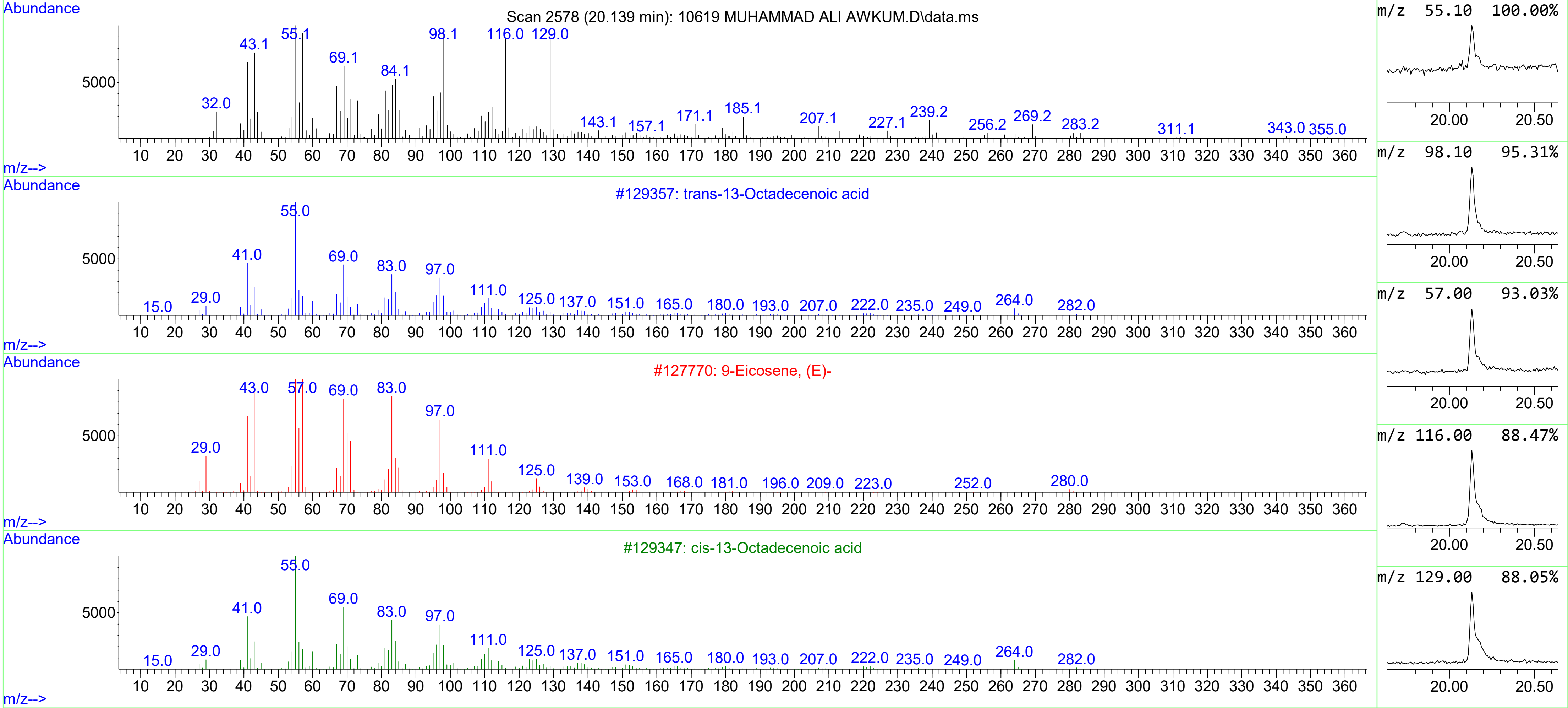

Data File: D:\GCMS RESULTS DATA\10619 MUHAMMAD ALI AWKUM.D
Sample : 6/1

Peak Number: 7 at 20.136 min Area: 5443593 Area % 2.62

The 3 best hits from each library.	Ref\#	CAS\#	Qual
C:\Database\NIST11.L			
1 trans-13-Octadecenoic acid	129357	000693-71-0	46
2 9-Eicosene, (E)-	127770	074685-29-3	44
3 cis-13-Octadecenoic acid	129347	013126-39-1	43

Unknown Spectrum based on Apex

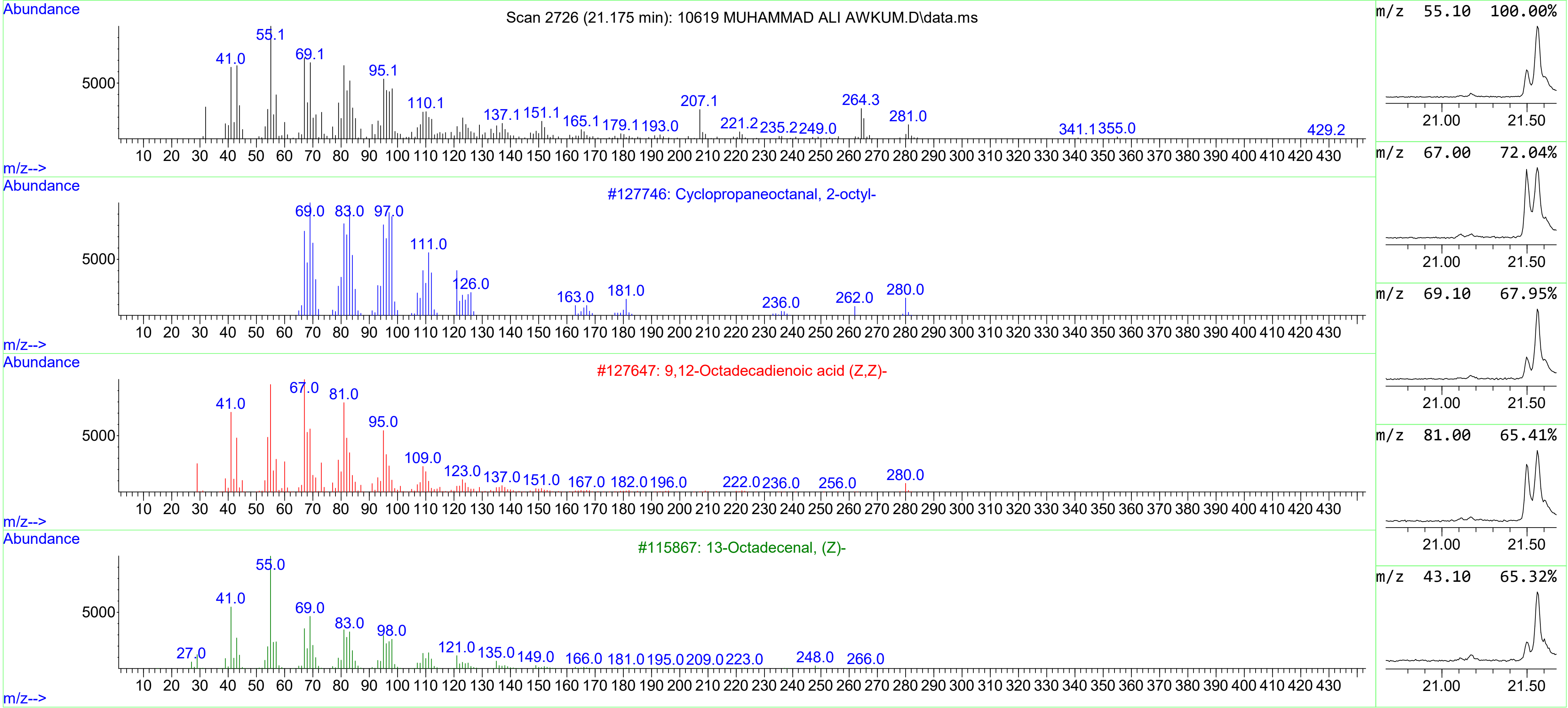


```
Data File: D:\GCMS RESULTS DATA\10619 MUHAMMAD ALI AWKUM.D
Sample   : 6/1

Peak Number:  8     at     21.174 min  Area:    2751829  Area % 1.32

 The 3 best hits from each library.          Ref\#    CAS\#      Qual
---------------------------------------------------------------------
    C:\Database\NIST11.L
     1 Cyclopropaneoctanal, 2-octyl-         127746 056196-06-6   96
     2 9,12-Octadecadienoic acid (Z,Z)-      127647 000060-33-3   96
     3 13-Octadecenal, (Z)-                  115867 058594-45-9   90
```

Unknown Spectrum based on Apex

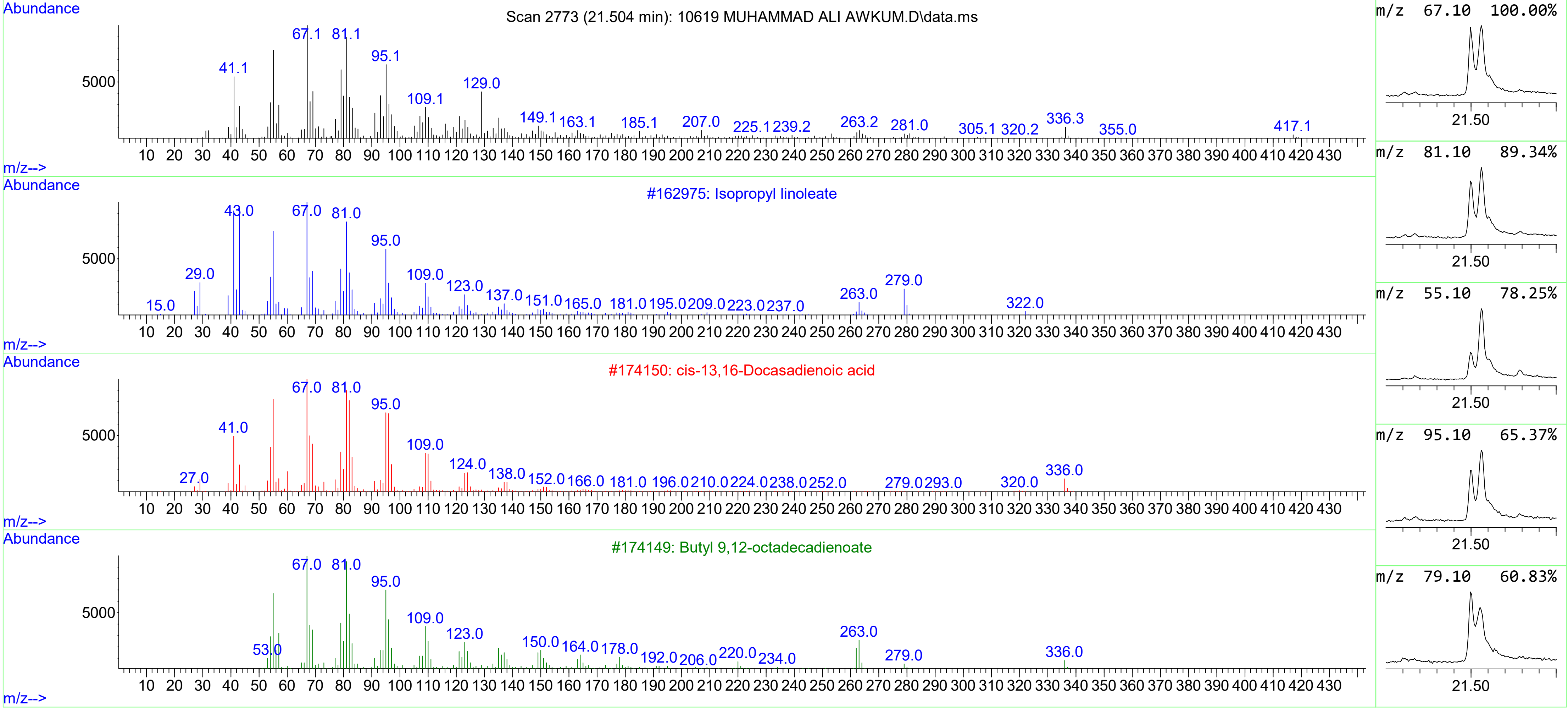

Data File: D:\GCMS RESULTS DATA\10619 MUHAMMAD ALI AWKUM.D
Sample : 6/1

Peak Number: 9 at 21.503 min Area: 17305197 Area % 8.33

The 3 best hits from each library.	Ref\#	CAS\#	Qual
C:\Database\NIST11.L			
1 Isopropyl linoleate	162975	022882-95-7	62
2 cis-13,16-Docasadienoic acid	174150	007370-49-2	58
3 Butyl 9,12-octadecadienoate	174149	1000336-54-1	55

Unknown Spectrum based on Apex

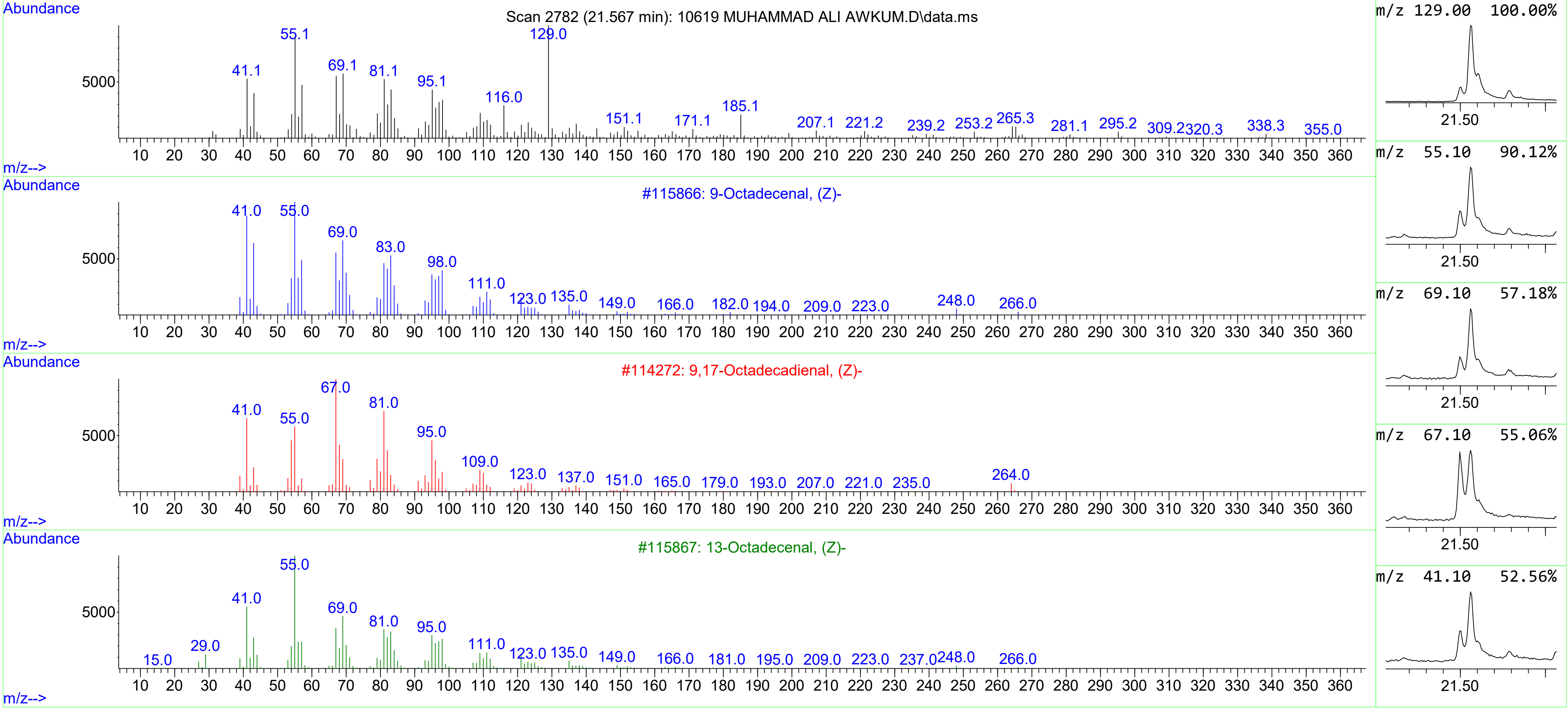

Data File: D:\GCMS RESULTS DATA\10619 MUHAMMAD ALI AWKUM.D
Sample : 6/1

Peak Number: 10 at 21.565 min Area: 60566769 Area % 29.15

The 3 best hits from each library.	Ref\#	CAS\#	Qual
C:\Database\NIST11.L			
1 9-Octadecenal, (Z)-	115866	002423-10-1	94
2 9,17-Octadecadienal, (Z)-	114272	056554-35-9	93
3 13-Octadecenal, (Z)-	115867	058594-45-9	81

Library Search Report - ChemStation Integrator

Unknown Spectrum based on Apex

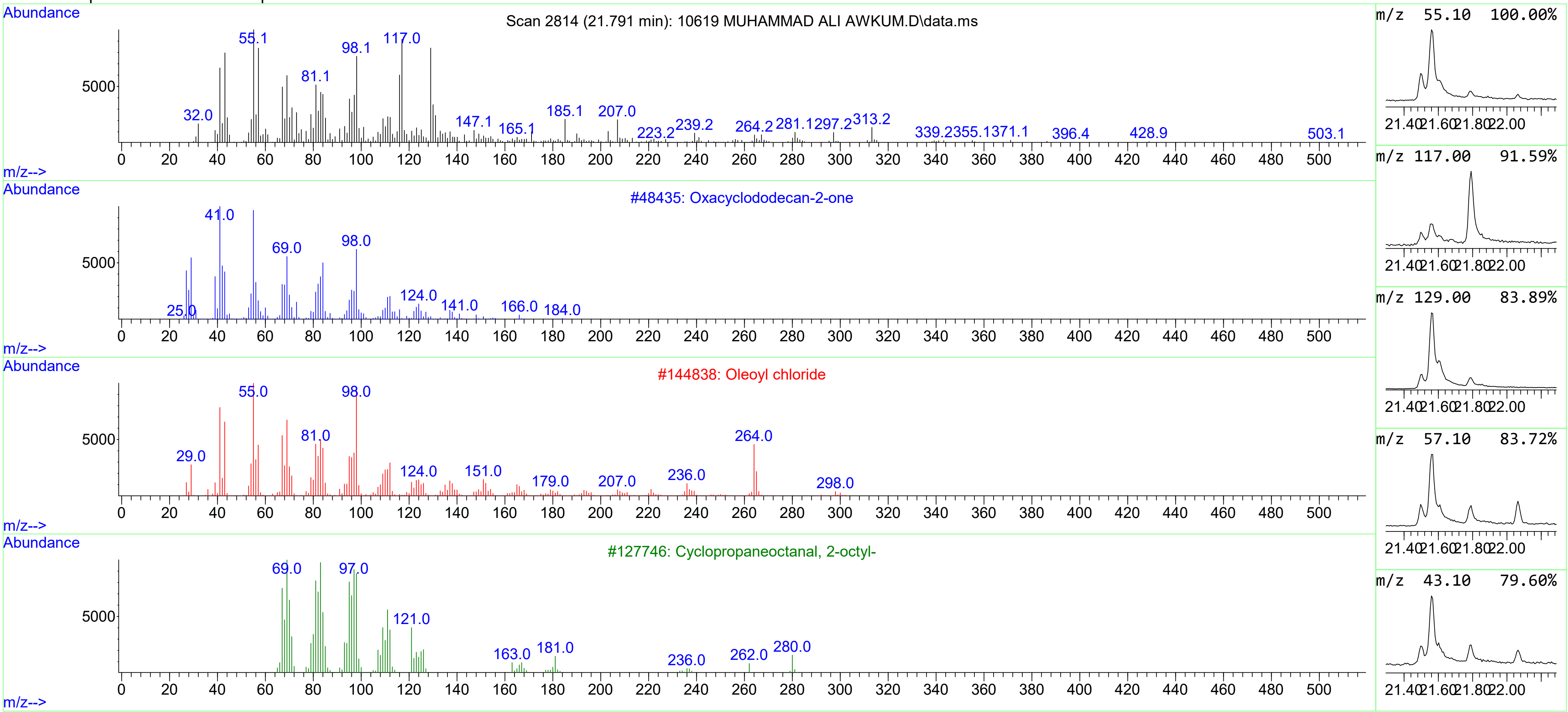


```
Data File: D:\GCMS RESULTS DATA\10619 MUHAMMAD ALI AWKUM.D
Sample   : 6/1

Peak Number:  11    at     21.792 min  Area:  10281472  Area % 4.95

 The 3 best hits from each library.          Ref\#    CAS\#      Qual
--------------------------------------------------------------------
    C:\Database\NIST11.L
     1 Oxacyclododecan-2-one                  48435 001725-03-7   51
     2 Oleoyl chloride                       144838 000112-77-6   46
     3 Cyclopropaneoctanal, 2-octyl-         127746 056196-06-6   45
```

Unknown Spectrum based on Apex

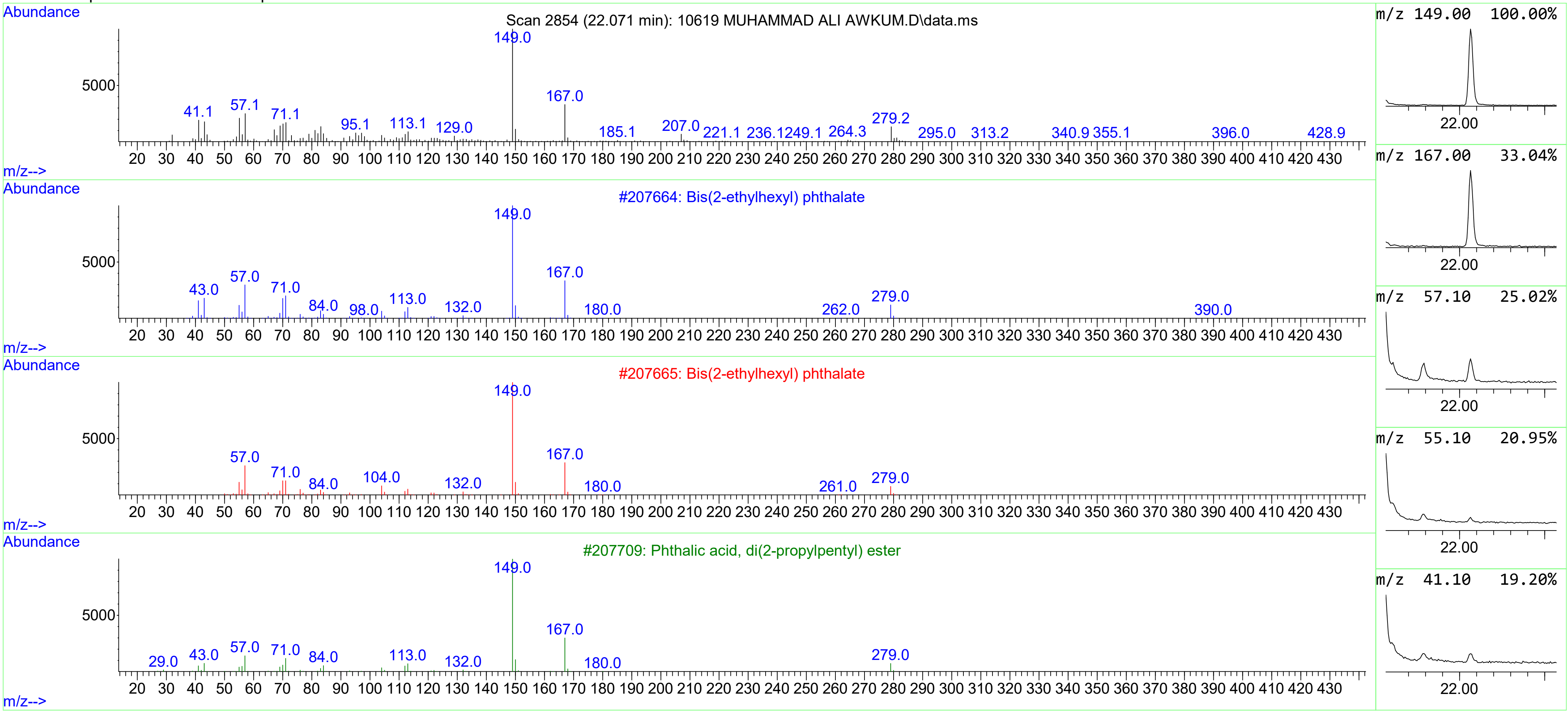

Data File: D:\GCMS RESULTS DATA\10619 MUHAMMAD ALI AWKUM.D
Sample : 6/1

Peak Number: 12 at 22.068 min Area: 5958527 Area % 2.87

The 3 best hits from each library.	Ref\#	CAS\#	Qual
C:\Database\NIST11.L			
1 Bis(2-ethylhexyl) phthalate	207664	000117-81-7	90
2 Bis(2-ethylhexyl) phthalate	207665	000117-81-7	86
3 Phthalic acid, di(2-propylpentyl...	207709	1000377-93-5	86

Unknown Spectrum based on Apex

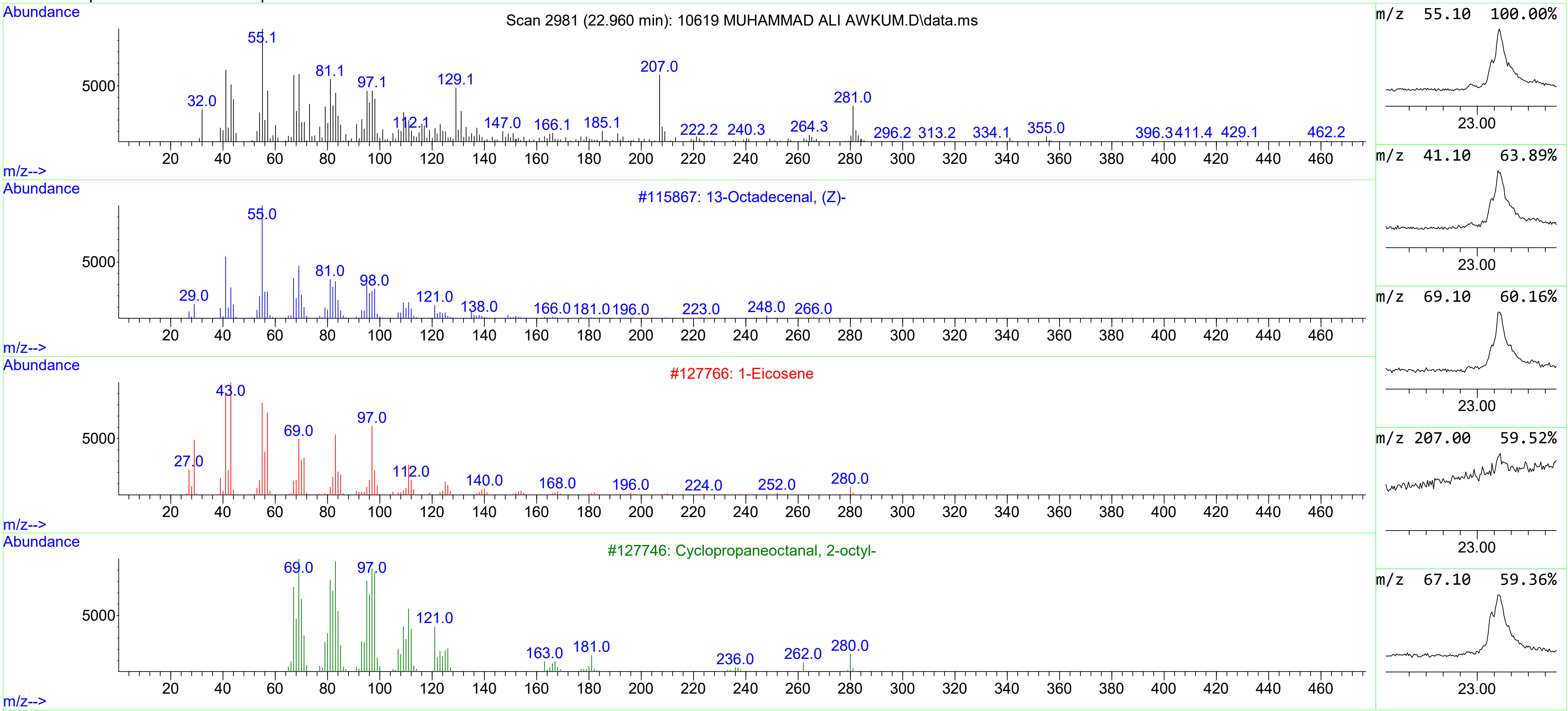

Data File: D:\GCMS RESULTS DATA\10619 MUHAMMAD ALI AWKUM.D
Sample : 6/1

Peak Number: 13 at 22.961 min Area: 1243422 Area % 0.60

The 3 best hits from each library.	Ref\#	CAS\#	Qual
C:\Database\NIST11.L			
1 13-Octadecenal, (Z)-	115867	058594-45-9	87
2 1-Eicosene	127766	003452-07-1	56
3 Cyclopropaneoctanal, 2-octyl-	127746	056196-06-6	55

Library Search Report - ChemStation Integrator

Unknown Spectrum based on Apex

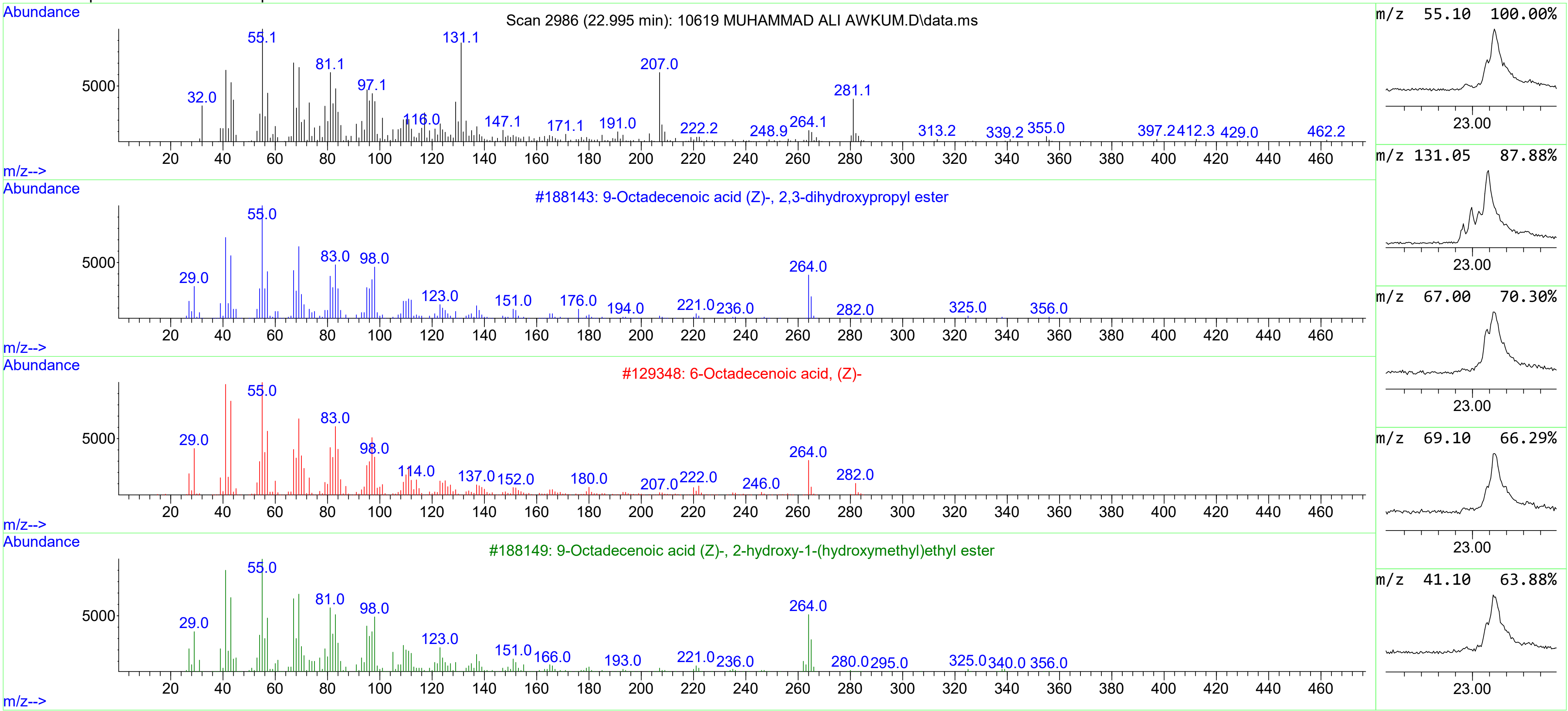

Data File: D:\GCMS RESULTS DATA\10619 MUHAMMAD ALI AWKUM.D
Sample : 6/1

Peak Number: 14 at 22.994 min Area: 1231046 Area % 0.59

The 3 best hits from each library.

C:\Database\NIST11.L

	The 3 best hits from each library.	Ref\#	CAS\#	Qual
1	9-Octadecenoic acid (Z)-, 2,3-di...	188143	000111-03-5	42
2	6-Octadecenoic acid, (Z)-	129348	000593-39-5	25
3	9-Octadecenoic acid (Z)-, 2-hydr...	188149	003443-84-3	20

Unknown Spectrum based on Apex

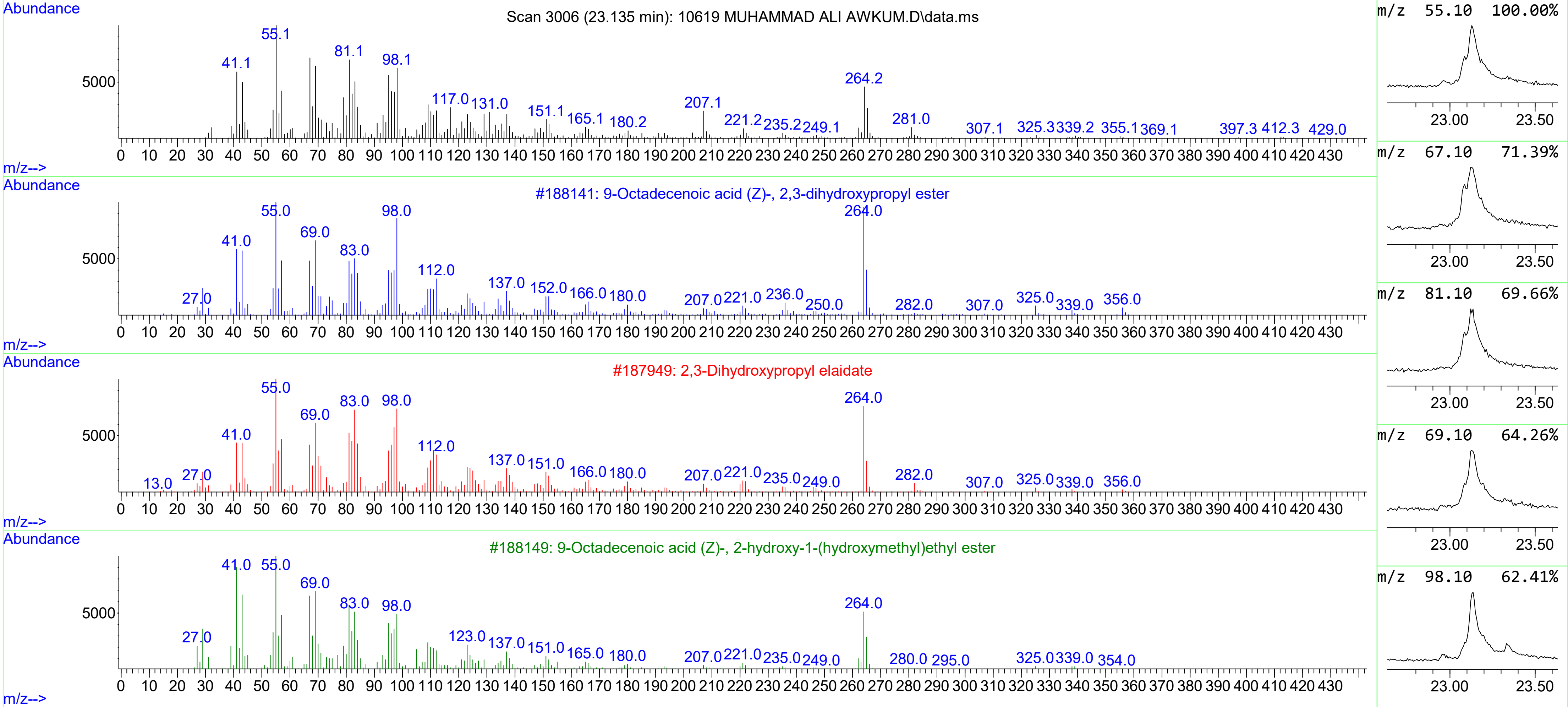

Data File: D:\GCMS RESULTS DATA\10619 MUHAMMAD ALI AWKUM.D
Sample : 6/1

Peak Number: 15 at 23.132 min Area: 54537893 Area % 26.25

The 3 best hits from each library.	Ref\#	CAS\#	Qual
C:\Database\NIST11.L			
1 9-Octadecenoic acid (Z)-, 2,3-di...	188141	000111-03-5	91
2 2,3-Dihydroxypropyl elaidate	187949	002716-53-2	91
3 9-Octadecenoic acid (Z)-, 2-hydr...	188149	003443-84-3	90

Unknown Spectrum based on Apex

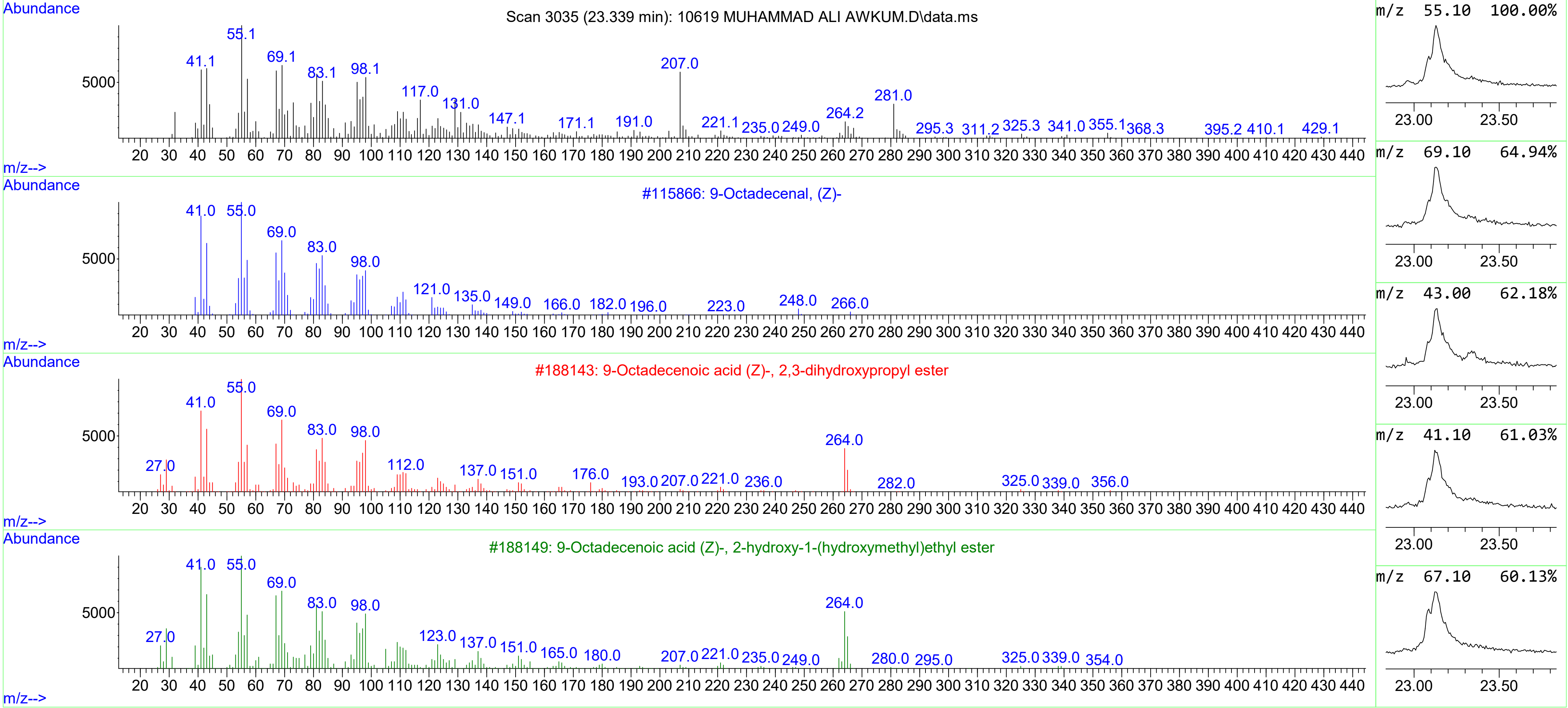

Data File: D:\GCMS RESULTS DATA\10619 MUHAMMAD ALI AWKUM.D
Sample : 6/1

Peak Number: 16 at 23.337 min Area: 9744104 Area % 4.69

The 3 best hits from each library.	Ref#	CAS#	Qual
C:\Database\NIST11.L			
1 9-Octadecenal, (Z)-	115866	002423-10-1	52
2 9-Octadecenoic acid (Z)-, 2,3-di...	188143	000111-03-5	43
3 9-Octadecenoic acid (Z)-, 2-hydr...	188149	003443-84-3	38

Library Search Report - ChemStation Integrator

Unknown Spectrum based on Apex

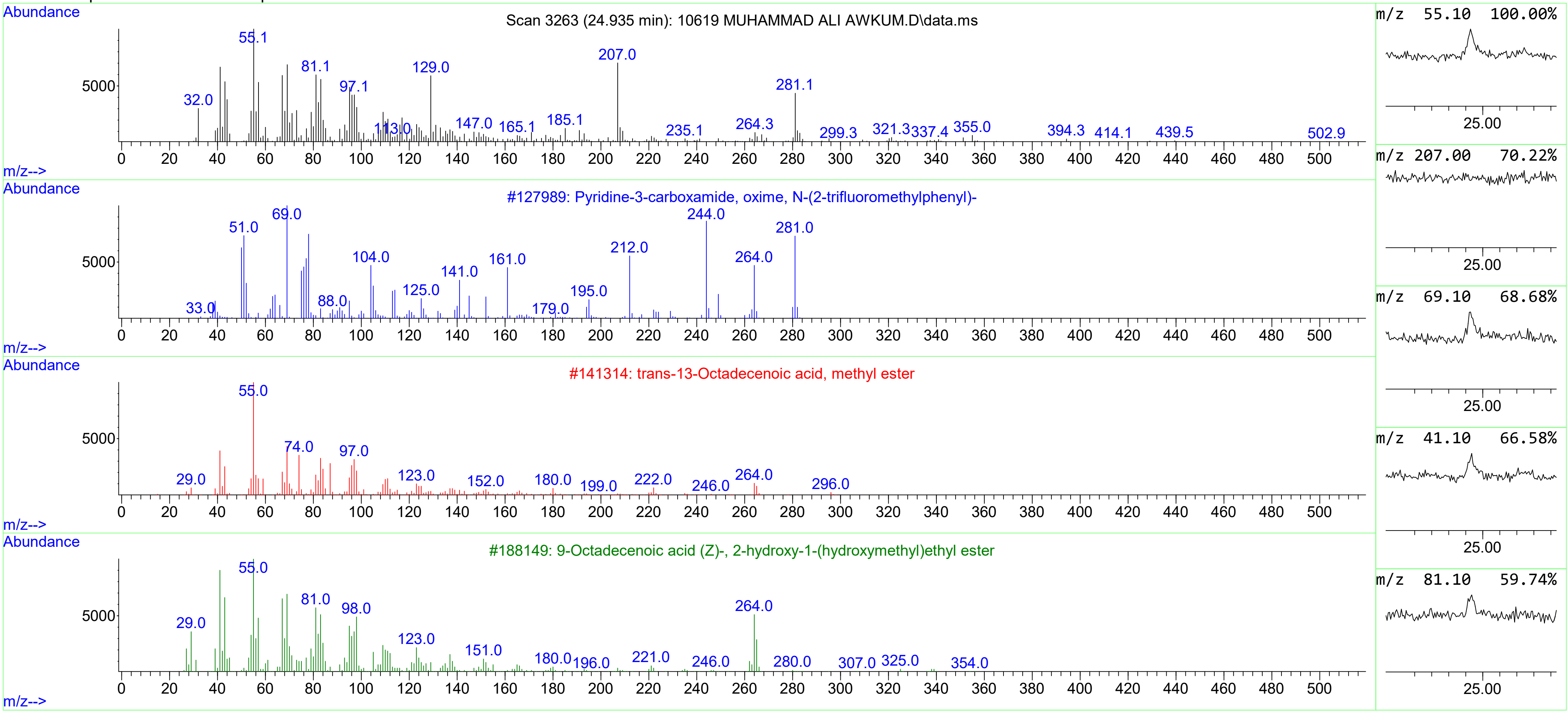

Data File: D:\GCMS RESULTS DATA\10619 MUHAMMAD ALI AWKUM.D
Sample : 6/1

Peak Number: 17 at 24.933 min Area: 2505994 Area % 1.21

The 3 best hits from each library.	Ref\#	CAS\#	Qual
C:\Database\NIST11.L			
1 Pyridine-3-carboxamide, oxime, N...	127989	288246-53-7	53
2 trans-13-Octadecenoic acid, meth...	141314	1000333-61-3	38
3 9-Octadecenoic acid (Z)-, 2-hydr...	188149	003443-84-3	38
